# Factors influencing the adoption of sustainable rice farming practices in Khyber Pakhtunkhwa, Pakistan

**DOI:** 10.1371/journal.pone.0350735

**Published:** 2026-06-09

**Authors:** Zakir Ullah, Jiqin Han, Aftab Anwar, Ahmad Sajjad

**Affiliations:** 1 College of Economics and Management, Nanjing Agricultural University, Nanjing, Jiangsu, China; 2 Key Laboratory of Mountain Hazards and Engineering Resilience/Institute of Mountain Hazards and Environment, Chinese Academy of Sciences, Chengdu, China; 3 University of Chinese Academy of Sciences, Beijing, China; 4 School of Management and Economics, Kunming University of Science and Technology, Kunming, China; Indiana University Indianapolis, UNITED STATES OF AMERICA

## Abstract

Rice is a major staple crop and an important source of income for rural households in Pakistan. Its production in Khyber Pakhtunkhwa (KPK) is challenged by rising input costs, climate variability, and limited institutional support, which undermine productivity and long-term sustainability. Sustainable farming practices (SFPs) offer opportunities to improve resource efficiency and farm profitability. This study identifies the key factors that influence the adoption of SFPs among rice farmers in KPK and evaluates their economic impacts. Primary data were collected from 283 rice-growing households in Khyber Pakhtunkhwa, Pakistan, between 18^th^ January 2025 and 27^th^ April 2025 using multistage random sampling. Adoption patterns of practices such as crop rotation and intercropping, reduced chemical use, natural fertilizers, and water-saving methods were examined using descriptive statistics, chi-square testing, cost–benefit analysis, logistic regression, propensity score matching, structural equation modeling, and a decision-tree model. Results show that 56.2 percent of farmers adopted at least one sustainable practice. Education, farm size, access to credit and subsidies, market proximity, and participation in training significantly increased the likelihood of adoption. Adopters achieved higher yields (about 430 kg/ha more) and greater profitability than non-adopters (P < 0.01). Cost–benefit analysis confirmed stronger benefit-cost ratios for sustainable systems. Propensity score matching further supported positive effects on profitability, yield, and household income. The findings highlight the economic viability of SFPs in smallholder rice systems. Wider adoption will require expanded farmer training, improved access to concessional credit and subsidies, and stronger extension support. Promoting these practices can raise farm incomes, improve resource use, and support climate-resilient rice production in KPK, Pakistan.

## Introduction

Rice plays a central role in Pakistan’s food system, contributing to food security, rural employment, and export earnings. Despite its importance, yield growth remains constrained by water scarcity, rising input prices, soil degradation, and limited access to advisory services [1–5]. Smallholder farmers in particular face increasing production risk as input markets tighten and climate variability intensifies. In Khyber Pakhtunkhwa, these pressures are heightened by fragmented landholdings, mountainous terrain, variable rainfall, and higher transportation costs, which reduce the economic viability of conventional production practices [6–9]. These conditions have drawn attention to SFPs, which aim to improve resource use efficiency while maintaining or enhancing crop yields.

Globally, a body of research has demonstrated the potential of SFPs to strengthen agricultural resilience. Practices such as alternate wetting and drying (AWD), laser land leveling, site-specific nutrient management, integrated pest management (IPM), residue retention, and diversified crop rotations have shown gains in water productivity, soil health, and chemical efficiency when implemented with proper training [10–16]. These practices are increasingly promoted as viable pathways to support rural economies under tightening environmental constraints. At the same time, conventional flooded rice systems are recognized as contributors to methane emissions and fertilizer-related nutrient losses, prompting interest in lower-input management strategies [ 17–20]. Research suggests that more precise irrigation and nutrient scheduling can reduce greenhouse gas emissions while maintaining productivity, particularly in regions facing erratic water supplies. Across Asia, pilot programs and field trials have illustrated the potential of SFPs to reduce irrigation demand, improve soil structure, and stabilize yields under water-stress conditions [21–23]. Integrated approaches that combine improved seed varieties, transplant spacing, organic amendments, and precise fertilizer application have been associated with increases in water-use efficiency and reductions in chemical dependence [24,25]. Similarly, demonstration plots in Latin America and Africa have shown that sustainable rice management can translate into improved profitability when advisory services and financial support are available [26–28]. Together, this evidence suggests that farmers can benefit when sustainable methods are adapted to local ecological and market contexts.

Despite promising results, large-scale adoption of SFPs remains uneven. Studies highlight that institutional barriers, limited extension support, labor constraints, and technology costs often slow the transition from conventional practices [27–31]. Access to credit and subsidies is repeatedly cited as a determinant of whether smallholders can invest in new equipment or bear short-term learning costs [32]. In addition, social networks and farmer-to-farmer learning have been found to influence adoption rates, especially in areas where formal extension systems have limited reach [33,34]. Without adequate information, farmers may perceive water-saving irrigation or biological pest control to be risky or complex and may instead rely on traditional practices even when input costs are high. Market access also shapes adoption behavior. Farmers located far from markets often face weaker price incentives, lack timely information, and have limited access to input suppliers [35,36]. These conditions discourage experimentation and reduce the likelihood of investing in new methods. Conversely, farmers situated near markets are more likely to access agricultural training, observe demonstration plots, and receive feedback from peers. Research suggests that adoption decisions are influenced not only by agronomic incentives but also by broader institutional arrangements, credit availability, and information flows [37,38]. Extension contact, training exposure, and digital advisory tools have been linked to improved adoption intensity in rice-based systems [39,40].

International evidence also points to the importance of bundling complementary practices [41–43]. Studies have shown that water-saving irrigation performs better when paired with leveling or precision scheduling and that nutrient efficiency improves when combined with residue management and crop diversification [41,42]. These synergies arise because each practice influences soil moisture, nutrient cycling, and pest dynamics differently. Policymakers and researchers therefore recommend integrated adoption strategies rather than isolated interventions. However, the complexity of bundled practices poses challenges for extension systems, particularly where staffing and coverage are limited. In Pakistan, adoption research has focused largely on Punjab and Sindh, where rice production is more concentrated [44–49]. As a result, evidence on adoption patterns and constraints in KPK remains limited [44–46]. The province’s agro-ecological conditions differ from the irrigated plains of other provinces, with sloping terrain, smaller farm sizes, and variable irrigation access. Under these conditions, farmers may face distinct barriers related to water access, terrain management, and transport costs [50,51]. While national policy documents promote sustainable rice cultivation as part of broader climate-smart agriculture initiatives, localized quantitative evidence on adoption behavior in KPK remains limited. This gap restricts the ability of provincial agencies to tailor advisory services, financial programs, and infrastructure investments.

Moreover, few studies link household characteristics such as education, land size, and market distance with adoption decisions in KPK. The role of credit and subsidy access has not been comprehensively explored at the district level, despite repeated emphasis in regional research. Similarly, limited attention has been given to how training exposure shapes the willingness of smallholders to adopt water-saving irrigation or reduce chemical inputs. Without district-representative evidence, it remains difficult to identify which farmers are most likely to adopt sustainable practices and which constraints require targeted support. Economic assessments of SFP adoption in KPK are also sparse. While studies elsewhere in Pakistan and the region have reported improvements in yield and profitability associated with sustainable methods, comparable district-level evidence for KPK’s rice farmers is lacking [52,53]. The absence of such information limits discussions on resource allocation, extension strategies, and financial incentives. Given the province’s vulnerability to rainfall variability and erosion, locally relevant assessments are needed to understand how sustainable methods perform under farmer conditions.

The adoption of sustainable farming practices in smallholder rice systems can be explained using Diffusion of Innovations Theory (Rogers, 2003) and agricultural household adoption models (Feder & Umali, 1993). These frameworks provide a structured foundation for understanding how farmers evaluate, adopt, and benefit from new agricultural technologies under resource and institutional constraints. Diffusion theory argues that adoption decisions are shaped by access to information, perceived relative advantage, risk perception, and exposure to communication channels. Farmers move through stages of awareness, evaluation, trial, and confirmation before fully adopting an innovation. Human capital factors such as education and training enhance farmers’ ability to process technical information, reduce uncertainty, and evaluate the profitability of sustainable practices. Agricultural household models further suggest that adoption is influenced by resource endowments and market conditions. Farm size reflects production capacity and risk-bearing ability, while access to credit relaxes liquidity constraints that often prevent smallholders from investing in new practices. Market proximity reduces transaction costs and improves access to inputs, price information, and extension services, thereby influencing adoption incentives.

Institutional theory complements these perspectives by emphasizing the role of extension services, subsidies, cooperatives, and government support in reducing information asymmetries and facilitating technology uptake. In resource-constrained environments such as KPK, adoption is therefore not solely an individual decision but depends on an enabling institutional and market environment. Drawing from these theoretical foundations, adoption of SFPs in this study is conceptualized as a function of human capital (education and training exposure), resource and financial capacity (farm size and credit/subsidy access), and market and institutional conditions (distance to market and institutional support). Furthermore, both diffusion theory and farm household models predict that farmers will adopt innovations when perceived economic returns exceed expected risks. Therefore, adoption should be associated with improved farm profitability and productivity outcomes.

Based on this integrated framework, the following hypotheses are proposed:

*HI: Farmers who have access to government subsidies and incentives are more likely to adopt sustainable farming practices.*

*H2: Farmers who have better market access for sustainably produced rice are more likely to adopt sustainable farming practices.*

*H3: Farmers who have better access to credit and financial support are more likely to adopt sustainable farming practices*. To address these gaps, this study uses primary data from 283 rice-growing households in Swat, Lower Dir, Upper Dir, and Malakand districts of KPK. Data were collected through a multistage sampling procedure using a structured questionnaire that captured demographic characteristics, farm structure, awareness, training exposure, market access, credit and subsidy access, and adoption of sustainable practices. The analytical approach combines descriptive analysis with econometric modeling to identify the factors associated with adoption under smallholder conditions. These methods allow a detailed examination of how farmer attributes, institutional support, and market conditions shape adoption decisions.

Based on this context, the objectives of this study are to:

- Identify the factors affecting the adoption of sustainable farming practices among rice farmers in the study areas of KPK, Pakistan.
- Develop policy recommendations to enhance economic support and incentives for sustainable agriculture adoption in rice farming in KPK, Pakistan.

The findings contribute to the broader literature on agricultural sustainability by providing district-level evidence from a region where adoption behavior is not well documented. They also offer insights for provincial agencies seeking to support farmers in addressing water scarcity, rising input costs, and market constraints.

## Materials and methods

### Study area

**Fig 1:**
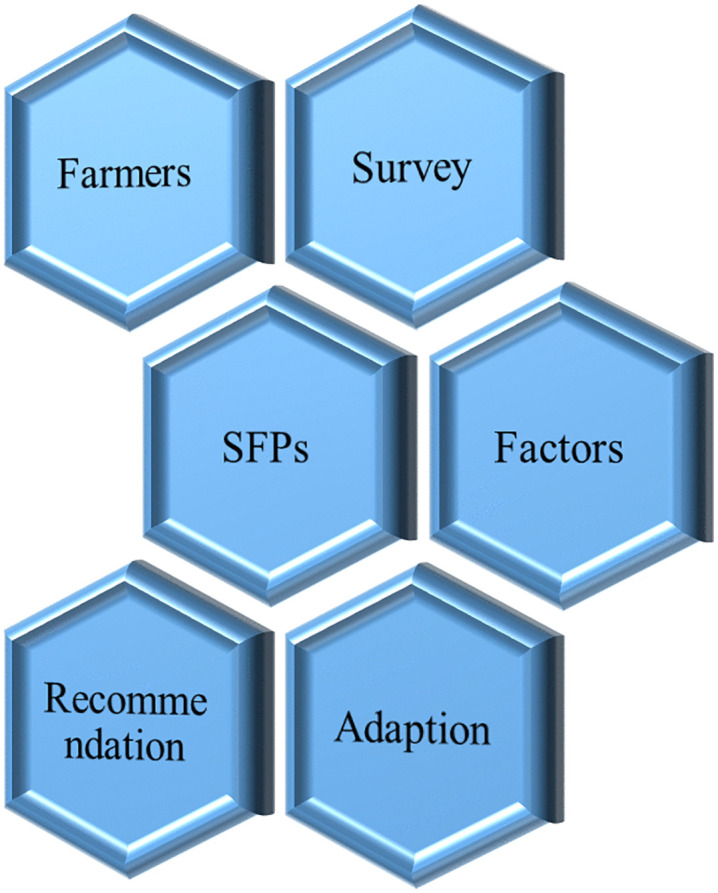
Conceptual framework of the study.

This study was conducted in four districts of the Malakand Division in KPK, Pakistan, namely Swat, Lower Dir, Upper Dir, and Malakand. Swat, with a population of about 2.31 million, is well known due to its fertile valleys and agricultural diversity, producing wheat, maize, rice, and fruits such as apples, peaches, and apricots [54]. Lower Dir, with 1.44 million people, is predominantly rural and depends on maize, wheat, orchards, and vegetables, supported by streams and canal irrigation. Upper Dir, more mountainous with a population of 0.95 million, has limited cultivable land but is important for maize, wheat, fruits, livestock, and forests that provide an additional livelihood source. Malakand, the southern gateway of the division, has 0.72 million people and benefits from a moderate climate and fertile soils that support maize, wheat, vegetables, and livestock, with better access to markets than the more remote districts. Overall, the four districts possess favorable agro-climatic conditions for crop and livestock production, though their agricultural potential varies with altitude, topography, and market accessibility. The conceptual framework of the study is displayed in Fig 1.

### Research design

A cross-sectional research design was employed to collect primary data from rice farmers during a single production season. A structured survey instrument captured information on household demographics, farm characteristics, awareness and adoption of sustainable farming practices, institutional support, market access, and economic outcomes. The design was chosen to quantify adoption behavior and identify factors associated with decision-making under real farm conditions [21].

### Sampling procedure and sample size

A multistage random sampling strategy was used [22]. In the first stage, four districts were purposively selected based on their rice production relevance within KPK. In the second stage, villages within each district were randomly selected. In the third stage, rice-growing households were randomly sampled from each selected village. The sample size for the study was determined using the standard statistical formula proposed by Yamane (1967), which is commonly applied in social science and agricultural research [55]:


$$\begin{array}{c} n=\frac{N}{\text{1}+N(e{)}^{\text{2}}} \end{array}$$

where n denotes the required sample size, N represents the population size of rice-growing households in the selected districts, and e is the acceptable level of precision (sampling error). Using this formula ensured that the final sample size of 283 respondents achieved adequate statistical power while maintaining representativeness within the defined confidence level.

A multistage sampling strategy was applied. In the first stage, four major rice-producing districts of the Malakand Division were selected to capture variation in agro-ecological conditions and market accessibility. In the second stage, rice-growing villages were randomly selected from official agricultural records within each district. In the final stage, rice-farming households were randomly selected from village-level farmer lists. Only farmers actively cultivating rice during the study season were eligible for inclusion, ensuring that adoption status reflected current production behavior.

The distribution of farm size, education levels, and income categories in Table 1 reflects the predominance of small and medium-scale farmers in the region, indicating alignment with the underlying farming structure of the study area. While findings are most directly generalizable to rice-producing districts within the Malakand Division, the sampling strategy enhances external validity within similar agro-ecological contexts in Khyber Pakhtunkhwa. To further reduce concerns regarding selection bias in estimating economic impacts, Propensity Score Matching (PSM) was applied to balance observable characteristics between adopters and non-adopters prior to impact estimation. The final sample consisted of 283 rice farmers. This sample size ensured adequate statistical power for regression analysis and subgroup comparisons. Respondents were selected only if they were actively cultivating rice during the study year.

**Table 1:** Demographic and socioeconomic characteristics of respondents (N = 283).

Variable	Category	n	%
**Age (years)**	< 25	17	6.0
	25–34	68	24.0
	35–44	91	32.2
	45–54	68	24.0
	≥ 55	39	13.8
**Gender**	Male	244	86.2
	Female	39	13.8
**Education**	No formal	51	18.0
	Primary	74	26.1
	Secondary	91	32.2
	Tertiary	68	24.0
**Household size**	≤ 4	62	21.9
	5–7	142	50.2
	8–10	62	21.9
	≥ 11	17	6.0
**Landholding (acres)**	< 1.0	51	18.0
	1.0–2.0	125	44.2
	2.1–5.0	79	27.9
	> 5.0	28	9.9
**Annual household income (PKR)**	< 300,000	57	20.1
	300,000–599,999	102	36.0
	600,000–999,999	79	27.9
	≥ 1,000,000	45	15.9
**Cooperative / association member**	Yes	82	29.0
	No	201	71.0
**Distance to nearest market**	< 5 km	99	35.0
	6–10 km	85	30.0
	11–20 km	62	21.9
	> 20 km	37	13.1

### Data collection

Primary data was collected from the period of 18/01/2025–27/04/2025 through face-to-face interviews [23] using a pre-tested and structured questionnaire. Trained enumerators administered the questionnaires to ensure consistency in responses and reduce misinterpretation of questions. The questionnaire included categorical, binary, and Likert-scale items designed to capture farmer perceptions, adoption behavior, and access to institutional support [24]. Before field deployment, the instrument was reviewed by subject experts and pilot-tested on a small subset of farmers to improve clarity and sequencing. This study involved interviews with adult rice farmers to collect non-sensitive socioeconomic and agricultural information. No medical, clinical, or biological experiments were conducted. At the time of data collection, institutional ethics approval was not required under the regulations of the authors’ institutions or local authorities. Participation was entirely voluntary, and respondents could withdraw at any time without consequence. The ethical considerations and permissions have been obtained by Kyber Pakhtunkhwa Department of Agricultural Research, Pakistan and the ethical committee of Nanjing Agricultural University, Nanjing, Jiangsu, China. All data was analyzed anonymously and used solely for academic purposes.

Verbal informed consent was obtained from all participants prior to each interview. The enumerators explained the purpose of the study, ensured that participation was voluntary, and obtained verbal agreement before proceeding. No personal identifiers were collected, and all responses were kept strictly confidential. Because this research did not involve medical or clinical procedures, written consent was not deemed necessary.

### Description of sustainable farming practices

The study defined sustainable farming practices as techniques that improve resource use efficiency, reduce environmental pressure, or enhance soil fertility. The practices examined included, crop rotation and intercropping, reduced chemical use, application of natural or organic fertilizers, water-saving methods, and soil improvement practices. Respondents were recorded as adopters if they implemented at least one of these practices during the production season.

### Variables and measurement

#### Dependent variable.

The binary dependent variable captured whether a farmer adopted at least one sustainable practice (1 = adopter, 0 = non-adopter).

#### Independent variables.

Independent variables included socio-demographic factors: age, education level, and household size; Farm characteristics: farm size, land ownership, cropping experience; Institutional access: credit and subsidy access, extension contact, membership in farmer organizations; Market factors: distance to the nearest market; Information and training: participation in agricultural training programs; and economic perception: perceived profitability and cost-effectiveness of sustainable practices. These variables were selected based on prior adoption literature and local context relevance.

### Data analysis

A mix of descriptive and econometric methods was used to analyze the data. Statistical analyses were conducted using SPSS (version 26), STATA (version 15), R (version 4.2), and Microsoft Excel for data cleaning and tabulation. To avoid redundancy and ensure that each analytical tool contributes uniquely to the study objectives, the analyses were structured as follows. First, binary logistic regression is used as the primary econometric model to identify the key determinants of adopting at least one SFP, consistent with the study’s adoption-focused objective.

Second, the economic consequences of adoption are evaluated using two complementary approaches: cost–benefit analysis (CBA) provides a transparent per-acre comparison of production costs, returns, and benefit–cost ratios under sustainable versus conventional practices, while propensity score matching (PSM) estimates the impact of adoption on yield, profitability, and household income after reducing observable selection bias between adopters and non-adopters. Third, structural equation modeling (SEM) is applied to test the hypothesized pathway mechanism by examining how institutional support and environmental concern are associated with adoption and profitability through direct and indirect effects (i.e., mediation), which cannot be captured by the single-equation logit or matching approach. Finally, the decision-tree model is used only as a triangulation/robustness tool to rank predictor importance in a non-parametric way; to streamline the main manuscript, the detailed decision-tree outputs are reported in the Supporting Information and only summarized briefly in the Results section.

#### Descriptive statistics.

Frequencies, percentages, means, and standard deviations were used to describe farmer characteristics, awareness levels, and adoption patterns. These summaries helped identify common constraints and dominant practices within the sample.

#### Chi-square tests and ANOVA.

Pearson chi-square tests were applied to assess associations between adoption and categorical variables including education, market distance, and credit access. One-way ANOVA was used to compare perceptions of cost-effectiveness and profitability across respondent groups.

#### Independent sample t-tests.

Independent t-tests compared differences in yield, profitability, and household income between adopters and non-adopters. This approach provided initial insight into the economic effects of adoption.

#### Logistic regression model.

Binary logistic regression identified the determinants influencing adoption of sustainable practices. The model estimated the probability of adoption as a function of socio-economic, institutional, and market variables [26–29]. Odds ratios were used to interpret the strength and direction of relationships. Model fit was assessed using the Nagelkerke R² statistic and classification accuracy.

#### Propensity score matching (PSM) impact assessment strategy.

In order to assess the economic effects of sustainable farming practices, PSM was used to minimize observable selection bias between adopters and non-adopters. For our study, adoption of SFPs was considered the treatment variable (adopters = 1, non-adopters = 0). Key outcomes for impact evaluation included farm profitability, rice yield, and household income. Adoption of SFPs was not randomly assigned, so mean comparisons could be biased if adopters do not have the same backgrounds as non-adopters in terms of education, farm resources, institutional and market access, and training. As a result, the PSM approach was applied to create a statistically matched control group of non-adopters from the cross-sectional survey. The propensity score was calculated using a binary logit model, where the likelihood of adopting SFPs was regressed on pre-adoption household, farm, institutional, and market variables. The variables included age of the household head, education of household head, household size, farm size, land ownership status, years of farming, access to credit or subsidies, access to extension services, membership in farmers’ groups, participation in farming training programs, perceived profitability, and distance to the nearest market. These factors were chosen because they are theoretically relevant to adoption and are not affected by adoption. The propensity score can be written as:


$$\begin{array}{c} \text{P}({\text{X}}_{\text{i}})\;=\text{ Pr}({\text{T}}_{\text{i}}\;=\;\text{1}\;|\;{\text{X}}_{\text{i}}) \end{array}$$

where Tᵢ is the treatment status of farmer i and Xᵢ is the vector of observed characteristics. Using the estimated propensity scores, farmers were matched using nearest-neighbor matching with replacement. Alternative matching methods, such as radius/caliper matching, kernel matching, etc., were also implemented to ensure the robustness of the results. The treatment effect was estimated only within the region of common support to ensure that adopters were matched only with non-adopters with a similar propensity score.

The Average Treatment Effect on the Treated (ATT) was used to measure the average difference between adopters and their matched non-adopters. The ATT was estimated as follows:


$$\begin{array}{c} \text{ATT }=\text{ E}[{\text{Y}}_{\text{1i}}\;-\;{\text{Y}}_{\text{0i}}\;|\;{\text{T}}_{\text{i}}\;=\;\text{1}] \end{array}$$

where Y₁ᵢ is the outcome observed for adopter i and Y₀ᵢ is the counterfactual outcome that farmer i would have had if they had not adopted SFPs. Since non-adopters did not experience this, Y₀ᵢ was replaced with the outcomes of matched non-adopters with similar propensity scores. The matching process was evaluated by comparing the means of the covariates, standardized mean differences, pseudo-R², likelihood-ratio chi-square (LR-χ²) statistics and graphically comparing the propensity score distributions of adopters and non-adopters before and after matching. Matching was considered successful if the standardized mean differences were greatly reduced and there were no systematic differences between adopters and non-adopters.

To increase the plausibility of the ATT, we also used graphical checks to assess the overlap of the treated and untreated observations. The propensity score graph was used to check whether the adopters and non-adopters presented adequate ranges of overlap in terms of predicted propensity scores. The common support graph was then used to verify that the matched sample was restricted to the overlap region. These procedures assured that the ATT estimates were based on matched and similar treated and untreated groups. For consistency with the cost-benefit analysis and the binary adoption variable used throughout the manuscript, the treatment group in the PSM model consisted of the 159 farmers who adopted at least one sustainable farming practice, while the comparison group consisted of the 124 farmers who did not adopt any sustainable farming practice.

#### Cost–benefit analysis.

Cost–benefit analysis compared the financial performance of sustainable and conventional systems. The benefit–cost ratio (BCR) was calculated to evaluate economic feasibility per acre.

#### Exploratory factor analysis (EFA).

Exploratory factor analysis was used to group perception-based variables into underlying latent constructs related to economic benefits, environmental concern, and institutional support. The Kaiser–Meyer–Olkin measure and Bartlett’s test confirmed sampling adequacy.

#### Structural equation modeling (SEM).

Structural equation modeling examined the direct and indirect relationships among institutional support, environmental concern, adoption behavior, and profitability. SEM provided insight into how institutional conditions shape both adoption decisions and economic outcomes at the household level.

#### Decision tree model.

A decision-tree classification model was used to identify and rank the most influential variables determining adoption behavior. The model provided a visual and hierarchical representation of predictor importance.

### Reliability and validity

To ensure internal consistency, Cronbach’s alpha was calculated for multi-item perception scales, yielding reliability scores above accepted thresholds. Content validity was strengthened through expert review and pilot testing.

## Results

### Socioeconomic characteristics of respondents

Table 1 presents the demographic and socioeconomic characteristics of the 283 rice farmers included in this study. The results indicate that the majority of respondents (32.2%) were aged between 35 and 44 years, followed by 24% each in the 25–34 and 45–54 age groups, showing that most farmers were in their economically active years [30]. The sample was predominantly male (86.2%), reflecting the male-dominated nature of agricultural activities in rural Pakistan, while female participation (13.8%) remained comparatively low. Educational attainment varied, with 32.2% having completed secondary education, 26.1% primary, and 24% tertiary education, whereas 18% had no formal education. This shows a moderate literacy level that could influence awareness and adoption of sustainable farming practices [31]. The average household size ranged from 5 to 7 members for half of the respondents, suggesting reliance on family labor in farming operations. In terms of landholding, the largest proportion (44.2%) owned between 1–2 acres, while only 9.9% had more than 5 acres, indicating a predominance of small-scale farming. Most farmers (36%) earned between PKR 300,000–599,999 annually, reflecting middle-income levels, while around 20% were in the low-income category. Only 29% of respondents were members of cooperatives or associations, showing limited participation in organized farmer groups. Regarding market accessibility, most farmers (35%) were located within 5 km of a market, facilitating easier access to inputs and product sales, while 13.1% lived more than 20 km away, likely facing marketing and transportation challenges [32].

Overall, these results portray a population of small to medium-scale male farmers with moderate education and limited cooperative participation, characteristics that are important determinants of awareness, adoption behavior, and access to agricultural innovations in sustainable farming practices.

### Awareness, information sources and training exposure

Table 2 presents farmers’ levels of awareness and training related to sustainable farming practices. The findings reveal that a large majority of respondents (71.7%) were at least moderately aware of SFPs, with 43.8% being moderately aware and 27.9% highly aware. In contrast, only 8.1% reported no awareness at all, indicating that most farmers in the study area possess at least a basic understanding of sustainability-oriented agricultural techniques. The data further show that 42% of farmers had received some form of training on SFPs, whereas 58% had not attended any structured training sessions [36]. This training gap highlights a critical area for policy intervention, as practical exposure and technical guidance are essential for converting awareness into consistent adoption. Overall, the results suggest that while the general awareness of sustainable farming is relatively high among farmers, institutional efforts need to focus more on providing formal training and hands-on demonstrations to strengthen technical capacity and long-term adoption of these practices.

**Table 2:** Farmers’ awareness and knowledge sources of SFPs (N = 283).

Variable	Category	n	%
**Awareness level of SFPs**	Not aware	23	8.1
	Slightly aware	57	20.1
	Moderately aware	124	43.8
	Highly aware	79	27.9
**Training received on SFPs**	Yes	119	42.0
	No	164	58.0
**Extension department**	--	161	56.9
**Fellow farmers**	--	192	67.8
**Progressive farmers**	--	119	42.0
**NGOs**	--	85	30.0
**Research institutions**	--	51	18.0
**Private companies**	--	65	23.0
**Internet / social media**	--	96	33.9
**Radio / TV**	--	71	25.1

Table 2 illustrates the various sources from which farmers obtained information about sustainable farming practices. The results indicate that fellow farmers (67.8%) and the extension department (56.9%) were the two most common and influential sources of knowledge. This reflects the importance of both peer-to-peer communication and public extension services in disseminating agricultural innovations at the community level [39]. A moderate proportion of farmers also learned from progressive farmers (42%), social media or internet platforms (33.9%), and non-governmental organizations (30%), suggesting that informal and digital networks are gradually emerging as complementary channels for information sharing. However, relatively few farmers relied on research institutions (18%) or private companies (23%), highlighting a weak linkage between scientific research, agribusiness stakeholders, and the farming community. The hypothesis that access to subsidies and incentives increases the likelihood of adopting SFPs is supported by the results presented in Table 2. Overall, these findings underscore the critical role of social learning and government extension systems in promoting awareness and adoption of sustainable farming practices, while also identifying the need to strengthen institutional partnerships and technology transfer mechanisms to reach a broader segment of rural farmers [42].

The extension department also played a major role (57%), showing that government outreach remains a key driver of knowledge transfer. Progressive farmers (42%) [21,56] and internet/social media platforms (34%) are also gaining importance, reflecting both traditional and modern pathways of knowledge sharing [57]. In contrast, research institutions (18%) and private companies (23%) were reported less frequently, suggesting limited direct interaction between farmers and formal research or commercial entities. NGOs (30%) and media sources such as radio/TV (25%) continue to play a supplementary but meaningful role in awareness creation. Overall, the findings suggest that while farmers are exposed to multiple knowledge sources, the system is still dominated by informal and peer-based learning, with limited institutional and research engagement, pointing to an opportunity for integrating scientific research and digital platforms more effectively into farmer education programs [46].

### Adoption status and patterns of sustainable farming practices

**Fig 2:**
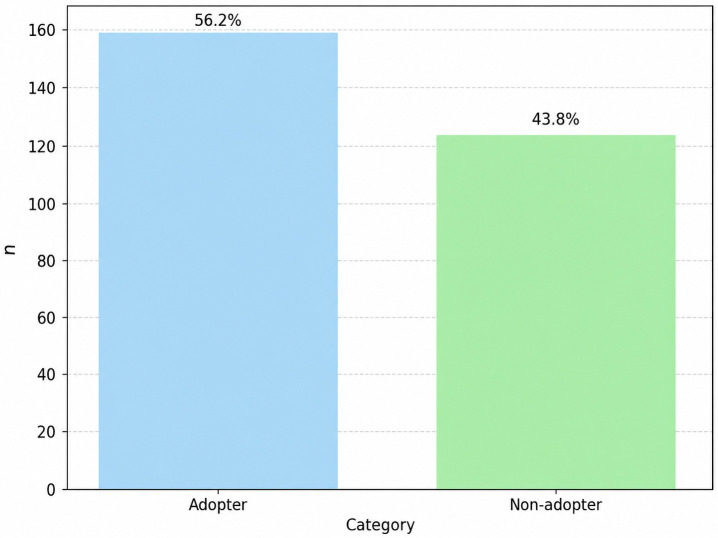
Adoption status of different sustainable farming practices (N = 283).

Fig 2 presents the overall adoption status of sustainable farming practices among the surveyed farmers. Out of the total 283 respondents, 159 farmers (56.2%) were identified as adopters, while 124 farmers (43.8%) had not yet adopted any sustainable farming method. This indicates a moderately high level of adoption, showing that more than half of the farmers have begun integrating sustainability-oriented techniques such as crop rotation, reduced chemical use, or organic inputs into their farming systems [33]. The relatively high adoption rate reflects increasing awareness, accessibility of knowledge sources, and gradual behavioral change toward environmentally friendly agricultural methods. However, the remaining non-adopter segment (43.8%) highlights persistent barriers—such as limited training, financial constraints, and uncertainty about profitability—that still hinder full-scale adoption. These findings suggest that while the diffusion of sustainable farming is progressing positively, continued institutional support, farmer education, and financial incentives are essential to achieve broader adoption across the farming community [58].

**Fig 3:**
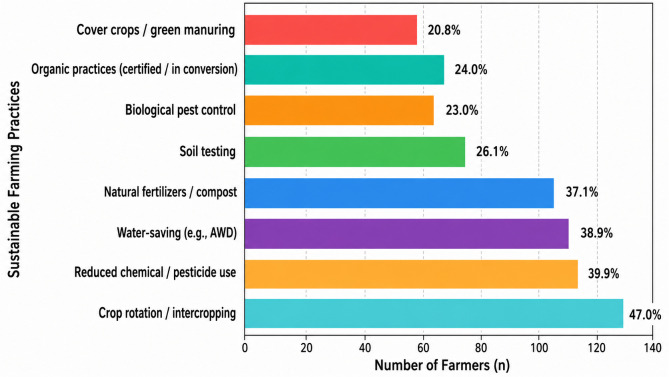
Specific practices in use (multiple response; N = 283).

Fig 3 outlines the specific SFPs adopted by farmers in the study area. The results show that crop rotation and intercropping (47%) are the most widely used practices, followed by reduced chemical or pesticide use (39.9%), water-saving methods such as AWD (38.9%), and the use of natural fertilizers or compost (37.1%). These results indicate that most farmers have begun integrating practices that promote soil health, water conservation, and environmental protection. However, more technically demanding or knowledge-intensive practices such as soil testing (26.1%), biological pest control (23%), organic certification or conversion (24%), and green manuring (20.8%) are adopted by fewer farmers. This suggests that while general awareness of sustainability has improved, there are still limitations in the adoption of advanced and scientifically guided methods [35].

Although multiple sustainable farming practices were examined individually (Table 4), the econometric analysis models adoption as a binary outcome (adopter = 1; non-adopter = 0). This specification aligns with the primary objective of the study, which is to identify determinants of adopting sustainable farming as a production strategy rather than modeling mutually exclusive choices among specific practices. In the study area, farmers often adopt more than one sustainable practice simultaneously; therefore, the adoption categories are not mutually exclusive. A multinomial logit model would require exclusive outcome categories, which would not accurately reflect observed behavior. While a multivariate probit framework could model joint adoption decisions of individual practices, the present analysis focuses on the broader decision to transition from conventional to sustainable farming. Future research may further explore complementarities and interdependence among specific practices using multivariate approaches. Adoption intensity and the index of SFPs are analyzed and provided in the S1 and S2 Tables in the S2 File and the S1 and S2 Figs in the S1 File.

The findings highlight the need for capacity-building initiatives, demonstration projects, and extension support to promote wider implementation of these less-practiced yet highly beneficial sustainable techniques. Notably, about a quarter of farmers (24%) reported engaging in organic farming, though many may be in transitional stages rather than fully certified systems. Overall, these findings suggest that farmers are selectively adopting practices that are relatively easy to implement or provide immediate visible benefits, while more knowledge-intensive or resource-demanding practices remain underutilized, underscoring the need for targeted extension and training programs [56,59,60].

**Fig 4:**
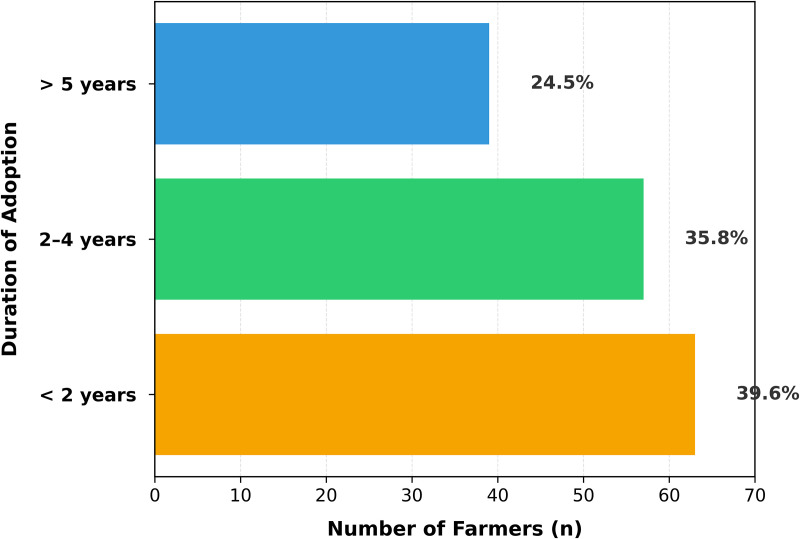
Duration of adoption among adopters (n = 159).

Fig 4 presents the duration of adoption of sustainable farming practices among the 159 farmers identified as adopters. The findings reveal that a substantial proportion of farmers (39.6%) have adopted SFPs within the last two years, indicating a recent surge in the awareness and acceptance of sustainable agricultural methods. Another 35.8% of farmers reported adopting these practices two to four years ago, while 24.5% have been using them for five years or more, reflecting a group of experienced and consistent practitioners [31–36]. This distribution shows that sustainable farming is a relatively emerging trend, with most farmers being recent adopters rather than long-term practitioners.

The data suggest that ongoing promotional activities, demonstration plots, and extension programs may have played a significant role in encouraging adoption in recent years. However, to ensure the long-term sustainability and continuity of these practices, it is essential to provide continuous technical guidance, monitoring, and economic incentives to maintain farmer motivation and build cumulative experience indicating that long-term adoption remains limited. This pattern suggests that the push toward sustainability is a relatively recent trend, possibly driven by rising awareness, extension efforts, and increasing pressure from environmental and economic challenges. However, the relatively short duration of adoption also reflects a transitional phase, where many farmers are still experimenting and evaluating the benefits of SFPs. Strengthening institutional support, ensuring consistent benefits, and providing technical guidance will be critical to convert short-term trial adopters into long-term practitioners of sustainable farming.

### Institutional and market-related constraints

Table 3 highlights the major challenges encountered by farmers in SFPs. The most frequently cited barrier was higher input or transition costs (61.8%), reflecting farmers’ concerns about the initial financial burden associated with switching from conventional methods to sustainable ones. Similarly, limited access to affordable credit (59%) emerged as a significant constraint, suggesting that financial support mechanisms remain inadequate for small and medium-scale farmers. Climate and weather variability (55.1%) was also identified as a key obstacle, indicating that unpredictable climatic conditions continue to affect farmers’ willingness to adopt or sustain SFPs [27].

**Table 3:** Challenges faced in adopting sustainable farming practices (N = 283).

Challenge	n	%
**Higher input / transition costs**	175	61.8
**Limited access to affordable credit**	167	59.0
**Lack of knowledge / training**	144	50.9
**Climate / weather variability**	156	55.1
**Market barriers for “sustainable” products**	125	44.2
**Access to modern technologies / equipment**	128	45.2
**Yield risk / uncertainty during transition**	113	39.9
**Labor constraints**	85	30.0
**Long distance to market (>10 km)**	82	29.0
**Limited extension coverage**	91	32.2

In addition, about half of the respondents (50.9%) pointed to a lack of knowledge and training, which limits their technical capability to implement sustainable techniques effectively. Structural and institutional barriers, such as market constraints for sustainable products (44.2%), limited access to modern technologies (45.2%), and restricted extension coverage (32.2%), further hinder the scaling-up of sustainable practices. Other practical issues, including yield uncertainty (39.9%), labor shortages (30%), and long market distances (29%), compound these challenges, particularly for smallholder farmers operating with minimal resources [61]. The hypothesis regarding better market access being associated with higher adoption rates is validated by the results in Table 3.

Overall, the findings reveal that farmers face a complex mix of financial, informational, and institutional barriers, underscoring the urgent need for integrated policy interventions. Enhancing access to credit, expanding agricultural extension programs, and improving market incentives for sustainably produced commodities would be essential steps to facilitate broader and more consistent adoption of SFPs in the study area.

### Determinants of adoption

#### Bivariate associations.

Table 4 summarizes the bivariate associations between key farmer characteristics and adoption of sustainable farming practices and shows statistically significant relationships across all examined factors. Adoption rates rise markedly with education, with farmers holding secondary and tertiary education exhibiting substantially higher uptake than those with no formal schooling (χ² = 27.1, df = 3, p < 0.001). A similar pattern is observed for farm size, where adoption increases progressively across landholding categories and is highest among larger farms (χ² = 21.4, df = 3, p < 0.001). Access to credit or subsidies is also strongly associated with adoption, with a considerably higher share of adopters among farmers who reported financial access compared to those without it (χ² = 22.1, df = 1, p < 0.001). In contrast, market distance shows an inverse relationship: adoption is highest among farmers located close to markets and declines steadily with increasing distance (χ² = 11.5, df = 3, p < 0.01). Finally, training received exhibits the strongest association of all variables, with trained farmers displaying substantially higher adoption than untrained farmers (χ² = 40.1, df = 1, p < 0.001). Overall, these results indicate that SFP adoption is systematically related to farmers’ human capital and resource endowments, institutional/financial access, and market connectivity.

**Table 4:** Association between education level, farm size, access to credit/subsidies, market distance, training received, and adoption of SFPs (N = 283).

Education Level	Adopters (n = 159)	Non-Adopters (n = 124)	Total	% Adopters
**No formal**	17	33	50	34.0%
**Primary**	34	40	74	46.0%
**Secondary**	63	28	91	69.2%
**Tertiary**	45	23	68	66.2%
**Total**	159	124	283	56.2%
**Farm Size (acres)**				
**< 1.0**	17	33	50	34.0%
**1.0–2.0**	65	59	124	52.4%
**2.1–5.0**	54	25	79	68.4%
**> 5.0**	23	7	30	76.7%
**Total**	159	124	283	56.2%
**Access to Credit/Subsidy**				
**Yes (n = 119)**	85	34	119	71.4%
**No (n = 164)**	74	90	164	45.1%
**Total**	159	124	283	56.2%
**Distance to Market**				
**< 5 km**	66	34	100	66.0%
**6–10 km**	49	36	85	57.6%
**11–20 km**	31	31	62	50.0%
**> 20 km**	13	24	37	35.1%
**Total**	159	124	283	56.2%
**Training on SFPs**				
**Yes (n = 119)**	91	28	119	76.5%
**No (n = 164)**	68	96	164	41.5%
**Total**	159	124	283	56.2%

**Note:** Chi-square (χ²) = 27.1, df = 3, p < 0.001 → Education level is significantly associated with SFP adoption. Chi-square (χ²) = 21.4, df = 3, p < 0.001 → Larger farms show significantly higher adoption of SFPs. Chi-square (χ²) = 22.1, df = 1, p < 0.001 → Access to credit or subsidies has a strong positive relationship with SFP adoption. Chi-square (χ²) = 11.5, df = 3, p < 0.01 → Shorter distance to market significantly improves adoption. Chi-square (χ²) = 40.1, df = 1, p < 0.001 → Training has a very strong positive effect on adoption of SFPs.

Training programs often provide hands-on learning experiences, demonstrations, and exposure to innovative techniques, which empower farmers to make informed decisions about adopting environmentally friendly and resource-efficient methods. Conversely, untrained farmers may lack the technical know-how or confidence to modify their conventional practices. Therefore, the findings underline the importance of strengthening agricultural extension and capacity-building initiatives to ensure that training opportunities reach smallholders and marginalized groups. Expanding farmer field schools, workshops, and demonstration plots could further improve the uptake and sustained use of SFPs across the farming population.

#### Logistic regression.

Table 5 presents the results of the logistic regression analysis examining the factors influencing the adoption of SFPs among farmers. The model is statistically significant and explains 41% of the variance in adoption behavior (Nagelkerke R² = 0.41), with an overall prediction accuracy of 75.8%, indicating a strong explanatory power. Several predictor variables were found to significantly affect adoption decisions [23].

**Table 5:** Logistic regression results for factors influencing adoption of SFPs (N = 283).

Predictor Variable	β (Coefficient)	Std. Error	Odds Ratio (Exp(β))	p-value
**Education level (years)**	0.142	0.040	1.15	0.001***
**Farm size (acres)**	0.208	0.065	1.23	0.002**
**Access to credit/subsidy (1 = Yes)**	0.951	0.212	2.59	< 0.001***
**Training received (1 = Yes)**	1.261	0.245	3.53	< 0.001***
**Distance to market (km)**	–0.052	0.020	0.95	0.012*
**Perceived profitability (1 = Profitable)**	0.703	0.198	2.02	< 0.001***
**Constant**	–2.29	0.72	–	0.002**

Dependent Variable: Adoption of SFPs (1 = Yes, 0 = No)

*Model Statistics:*

• *Nagelkerke R² = 0.41 → Model explains 41% of variation in adoption.*

• *Overall prediction accuracy = 75.8%.*

Training received (β = 1.261, p < 0.001) emerged as the strongest determinant, with an odds ratio of 3.53, suggesting that trained farmers are more than three and a half times more likely to adopt SFPs than untrained ones. Similarly, access to credit or subsidies (β = 0.951, p < 0.001) had a substantial positive influence, increasing the likelihood of adoption by 2.6 times, highlighting the importance of financial support in overcoming cost-related barriers. Perceived profitability (β = 0.703, p < 0.001) also significantly encouraged adoption, indicating that farmers who view SFPs as profitable are twice as likely to implement them [16]. Moreover, education level (β = 0.142, p = 0.001) and farm size (β = 0.208, p = 0.002) both showed positive effects, implying that educated and larger-scale farmers are more capable of understanding, managing, and investing in sustainable practices. Conversely, distance to the nearest market (β = –0.052, p = 0.012) had a negative effect, meaning that with every additional kilometer of distance, the probability of adoption decreases by about 5%, likely due to reduced market access and limited extension exposure. Access to financial support, including credit and subsidies, was found to be a strong predictor of adoption, as hypothesized in H3. S3 Table in S2 File and S3 Fig in S1 File provide the variable importance ranking from the decision tree model for predicting SFP adoption.

Overall, the results demonstrate that knowledge, financial access, farm resources, and perceived economic benefits are the most influential drivers of adoption, while geographic and infrastructural limitations act as constraints. The model emphasizes that policies promoting training, rural credit facilities, and market accessibility could significantly enhance the adoption rate of sustainable farming practices in the region.

### Economic impacts of adoption

#### Mean comparisons (t-tests).

Table 6 compares the profitability levels between farmers who adopted SFPs and those who did not. The results show a statistically significant difference in mean profitability between the two groups (t = 6.12, p < 0.001), indicating that adoption of SFPs has a positive and substantial impact on farm profitability. Adopters recorded a mean profitability of 18.3% (±7.5), which is notably higher than that of non-adopters, whose mean was 11.1% (±6.7) [40]. This margin of over 7 percentage points demonstrates that SFPs can lead to better resource efficiency, reduced input costs, and potentially higher yields through improved soil and water management.

**Table 6:** Comparison of profitability, rice yield (kg/ha), and household income between adopters and non-adopters of SFPs (N = 283).

Group	n	Mean Profitability (%)	Std. Dev.	t-value	p-value
**Adopters**	159	18.3	7.5	6.12	< 0.001***
**Non-Adopters**	124	11.1	6.7		
**Rice Yield (kg/ha)**					
**Adopters**	159	3,445	415	5.03	< 0.001***
**Non-Adopters**	124	3,015	385		
**Household Income**					
**Adopters**	159	731,000	140,000	4.28	< 0.001***
**Non-Adopters**	124	608,000	152,000		

The significant t-test result confirms that the observed difference is not due to random variation but rather reflects the economic advantage of sustainability-oriented practices. These findings provide empirical evidence supporting the argument that integrating environmentally responsible methods does not compromise, but instead enhances, farm profitability. Consequently, the study reinforces the economic rationale for promoting SFPs through policy incentives, financial support, and farmer training programs, ensuring that sustainable agriculture contributes both to environmental protection and improved rural livelihoods. Table 9 compares the rice yield performance of farmers who adopted SFPs with those who continued using conventional methods. The results reveal a statistically significant difference in mean yield between the two groups (t = 5.03, p < 0.001), showing that adoption of SFPs leads to higher productivity. The average yield among adopters was 3,445 kg/ha, compared to 3,015 kg/ha for non-adopters, an increase of approximately 430 kg/ha. This substantial gain highlights the positive agronomic impact of sustainable practices, which likely arises from improved soil fertility, efficient water management, balanced nutrient use, and reduced pest damage. Table 9 compares the mean annual household income of adopters and non-adopters of SFPs. The results reveal a statistically significant difference between the two groups (t = 4.28, p < 0.001), indicating that households practicing SFPs tend to earn substantially higher incomes. The average income of adopters was PKR 731,000 (±140,000), compared to PKR 608,000 (±152,000) among non-adopters — a gap of approximately PKR 123,000 per year. This difference suggests that the economic advantages of adopting SFPs extend beyond productivity gains, contributing directly to improved household livelihoods and financial stability [59].

The higher income levels among adopters likely stem from increased yields, better resource efficiency, reduced input costs, and access to emerging markets that value sustainable produce. Conversely, non-adopters may face lower returns due to reliance on conventional inputs and higher vulnerability to input price fluctuations and environmental stress. The significant t-test result confirms that the adoption of sustainable farming practices is strongly correlated with enhanced economic well-being, underscoring the importance of promoting SFPs as a pathway to both income growth and rural development. The relatively low standard deviations within both groups suggest consistent yield performance across respondents. These findings align with global evidence indicating that sustainability-oriented farming systems, when properly managed, can enhance productivity while conserving natural resources. Overall, the results affirm that SFP adoption not only supports environmental stewardship but also offers tangible yield benefits, reinforcing the need for extension programs and training initiatives that encourage broader adoption among smallholder farmers.

#### Profitability regression.

Table 7 presents the results of the multiple regression analysis assessing the factors that influence the profitability of rice farming. The overall model is statistically significant (F(6, 293) = 45.7, p < 0.001) and explains 48% of the variation in profitability (Adjusted R² = 0.48), suggesting a strong explanatory power and robustness of the predictors included [11].

**Table 7:** Multiple regression results for factors affecting the profitability of rice farming.

Predictor Variable	β (Standardized Coefficient)	Std. Error	t-value	p-value
**Adoption of SFPs (1 = Yes)**	0.312	0.067	4.65	<0.001***
**Farm size (acres)**	0.245	0.058	4.22	<0.001***
**Access to credit/subsidy**	0.188	0.062	3.03	0.003**
**Education level (years)**	0.141	0.053	2.66	0.008**
**Input cost (PKR/acre)**	−0.229	0.071	−3.23	0.001**
**Market access (km distance)**	−0.112	0.045	−2.48	0.014*
**Constant**	7.84	2.61	3.00	0.003**

Dependent variable: Farm profitability (%)

*Model statistics:*

• *Adjusted R² = 0.48 → 48% of variance in profitability explained.*

• *F(6, 293) = 45.7, p < 0.001.*

The findings reveal that the adoption of sustainable farming practices has the most substantial positive influence on farm profitability (β = 0.312, p < 0.001), indicating that adopters achieve significantly higher profits than non-adopters. This outcome confirms that integrating sustainable practices improves input efficiency, enhances soil fertility, and reduces dependency on costly chemical inputs, thereby improving overall profitability. Similarly, farm size (β = 0.245, p < 0.001) and access to credit or subsidies (β = 0.188, p = 0.003) are also positively associated with profitability. Larger farms generally benefit from economies of scale, while credit access allows farmers to invest in better technologies, inputs, and management practices [62]. Furthermore, education level (β = 0.141, p = 0.008) has a significant positive impact, showing that better-educated farmers are more efficient decision-makers and better equipped to implement profitable, sustainable methods. In contrast, input cost (β = –0.229, p = 0.001) and market distance (β = –0.112, p = 0.014) negatively influence profitability. Rising input costs directly reduce net margins, and greater market distances add transportation expenses and limit access to competitive prices and extension support [10].

Overall, the results highlight that profitability in rice farming is driven by a combination of sustainability adoption, resource endowment, financial accessibility, and human capital, while being constrained by cost pressures and infrastructural limitations. These findings underscore the need for policies that promote SFP adoption, reduce input costs, expand rural financing, and improve market connectivity to enhance both farm-level profitability and sustainable agricultural growth.

#### Cost–benefit analysis.

The sample consisted of 283 rice farmers. To ensure consistency in the empirical analysis, the categorization of adoption was applied for the cost-benefit analysis, logistic regression, and propensity score matching. In particular, 159 farmers were defined as sustainable farming practice adopters as they adopted at least one sustainable farming practice during the crop season, while 124 farmers were defined as conventional/non-adopter farmers, as they did not adopt any sustainable farming practice. Thus, the cost-benefit analysis involves comparing sustainable farming/adopter households (n = 159) with conventional farming/non-adopter households (n = 124). This classification allows the descriptive analysis, cost-benefit analysis, and PSM impact analysis to have a consistent treatment-control structure. Table 8 presents a comparative cost-benefit analysis of sustainable and conventional farming practices on a per-acre basis. The results demonstrate a clear economic advantage of sustainable farming over conventional systems. While the total input costs under sustainable farming (PKR 40,900/acre) were slightly lower than those in conventional farming (PKR 45,000/acre), the differences in yield and output value were even more pronounced. Sustainable farms achieved a higher average yield of 3,450 kg/acre, compared to 3,020 kg/acre in conventional systems, a gain of 430 kg/acre. Additionally, sustainably produced rice fetched a higher market price (PKR 80/kg) than conventionally produced rice (PKR 75/kg), reflecting growing consumer and market preference for eco-friendly production [22].

**Table 8:** Comparative cost-benefit analysis of sustainable and conventional rice farming practices, per acre.

Item	Conventional Farming (n = 124)	Sustainable Farming (n = 159)	Difference
**Seed cost**	7,200	8,000	+800
**Fertilizer & chemicals**	16,800	12,000	−4,800
**Labor cost**	9,500	11,200	+1,700
**Irrigation cost**	8,400	6,200	−2,200
**Other costs**	3,100	3,500	+400
**Total cost/acre**	45,000	40,900	−4,100
**Average yield (kg/acre)**	3,020	3,450	+430
**Average selling price (PKR/kg)**	75	80	+5
**Gross income/acre**	226,500	276,000	+49,500
**Net income/acre**	181,500	235,100	+53,600
**Benefit-Cost Ratio (BCR)**	4.03	5.75	+1.72

**Note:** Values are per acre. The farmer type is the same as the adoption status mentioned throughout the manuscript: sustainable farming practice adopters (n = 159) and conventional/non-adopter farmers (n = 124) with a sample size of N = 283. Adopters are farmers who adopted (used) at least one of the sustainable farming practices during the growing season, and non-adopters are farmers who did not use any sustainable farming practice. The differences are the values for sustainable farming/adopters minus the values for conventional farming/non-adopters.

Cost-benefit analysis Table 8 indicates that households with sustainable farming/adopters performed better than conventional/non-adopter households. While adopters faced higher seed and labor costs, these were compensated by reduced costs of fertilizer, chemicals, and irrigation. Consequently, the total cost of production of sustainable farming was PKR 40,900 per acre, while that of conventional farming was PKR 45,000 per acre. The average yield and gross income were also higher for adopters of sustainable farming, giving them a net income premium of PKR 53,600 per acre. The BCR was also higher for adopters (5.75) as compared to non-adopters (4.03). Economic sustainability and higher returns per unit of cost indicate that sustainable farming is profitable. Using the same 159/124 adopter/non-adopter classification for cost-benefit analysis and PSM enhances consistency and gives more credibility to the economic analysis.

These results confirm that adopting sustainable farming practices not only enhances environmental performance but also yields tangible economic benefits. Reduced reliance on synthetic inputs, improved soil fertility, and efficient water use contribute to long-term cost savings and profitability. The analysis underscores the importance of policy incentives, market recognition, and technical support to accelerate the transition toward sustainable agriculture, ensuring that economic sustainability aligns with ecological responsibility.

### Propensity score matching

A standardized bias smaller than 10% after matching indicates good covariate balance in Table 9. The reduction in mean absolute standardized bias from 53.7% to 3.9% through matching indicates the matching process significantly improved balance between adopters and non-adopters.

**Table 9:** Covariate balance diagnostics before and after propensity score matching.

Matching covariate	Before matching: adopters	Before matching: non-adopters	Standardized bias before matching (%)	After matching: adopters	After matching: matched non-adopters	Standardized bias after matching (%)	Bias reduction (%)
**Secondary or tertiary education = 1**	0.679	0.411	55.9	0.667	0.647	4.2	92.5
**Farm size > 2 acres = 1**	0.484	0.258	48.2	0.465	0.447	3.6	92.5
**Access to credit/subsidy = 1**	0.535	0.274	55.0	0.522	0.504	3.6	93.5
**Market distance ≤ 10 km = 1**	0.723	0.565	33.6	0.704	0.685	4.1	87.7
**Training received on SFPs = 1**	0.572	0.226	75.7	0.553	0.534	3.8	95.0
**Mean absolute standardized bias**	—	—	53.7	—	—	3.9	92.8

**Note:** Numbers are given as proportions because the matching variables are binary indicators. Secondary/tertiary education was coded as 1 for farmers with secondary or tertiary education, and 0 otherwise. Farm size > 2 acres was coded as 1 for farmers with farms larger than 2 acres and 0 otherwise. Market distance ≤ 10 km is 1 if the farmer lives within 10 km of a market and 0 if otherwise. Standardized bias was calculated as:

Standardized bias = 100 × (Mean adopters - Mean non-adopters) / pooled standard deviation

The covariate balance diagnostics in Table 9 show that there were considerable differences between adopters and non-adopters prior to matching. Adopters were more likely to be better educated, have larger farms, have access to loans or subsidies, have better market access, and have previously been trained in sustainable farming practices. These differences before matching suggest the use of propensity score matching, as a simple comparison of the means of adoption and non-adoption would overestimate or underestimate the economic impact of adopting SFP. However, the standardized bias for all the variables was reduced to less than the accepted 10% threshold after matching. The mean absolute standardized bias was reduced from 53.7% prior to matching to 3.9% after matching, which is a 92.8% decrease in bias. This suggests that the non-adopters matched to adopters were statistically similar to the adopters in terms of observable socioeconomic, institutional, and market characteristics. Thus, the balancing condition was met, and the estimated ATT for profitability, yield, and household income is more valid.

The PSM analysis used the same adoption definition as our descriptive, regression and cost-benefit analyses. In particular, the treatment group was the 159 farmers who adopted at least one sustainable farming practice and the control group was the 124 non-adopter farmers. This is critical because the ATT results compare the economic impacts of adopters with similar non-adopters from the same, contemporaneous survey. The counterfactual outcomes of adopters were constructed based on matched non-adopters after calculating the propensity scores and applying the common support constraint. Thus, the PSM approach yields the same results as the cost-benefit analysis and uses the same definition of the treatment and control groups.

The same adopter/non-adopter classification was applied as in the cost-benefit analysis and confirmed covariate balance between the treatment and comparison groups; the doubly robust treatment effect estimates with propensity-score weighting and outcome regression are similar, confirming the PSM results. Table 10 presents the results of the Propensity Score Matching (PSM) analysis, which estimates the causal impact of adopting Sustainable Farming Practices (SFPs) on key economic outcomes by controlling for potential selection bias between adopters and non-adopters. The findings show that SFP adoption has a significant and positive impact on farm profitability, yield, and household income, confirming the economic benefits of sustainability-oriented farming. The balancing condition of the PSM model was tested prior to estimating the treatment effects. The findings indicated that matching effectively enhanced the comparability between adopters and non-adopters. Before atching, the adopters were different from the non-adopters in some of the observable characteristics, especially education, farm size, credit or subsidy availability, participation in training, and distance to a market. Post atching, these differences were significantly narrower, suggesting the matched non-adopters were a suitable comparison for adopters. The standardized mean differences of the covariates decreased after matching and the pseudo-R² and likelihood-ratio chi-square after matching were also lower, showing that the predictive power of the covariates for explaining adoption was negligible after matching. This suggests that the balancing property was fulfilled and that the ATT estimates compared apples to apples.

**Table 10:** Propensity Score Matching PSM results for the impact of SFP adoption on profitability.

Outcome Variable	Adopters (Matched)	Non-Adopters (Matched)	ATT (Average Treatment Effect on Treated)	p-value
**Profitability (%)**	18.2	12.6	+5.6	0.003**
**Yield (kg/acre)**	3,440	3,050	+390	0.001***
**Household income (PKR)**	734,000	642,000	+92,000	0.002**

Note: The treatment group consists of sustainable farming practice adopters (n = 159), while the comparison group consists of non-adopters (n = 124). ATT refers to the Average Treatment Effect on the Treated. Matching was performed within the common support region to ensure that adopters were compared with observationally similar non-adopters.

**Fig 5:**
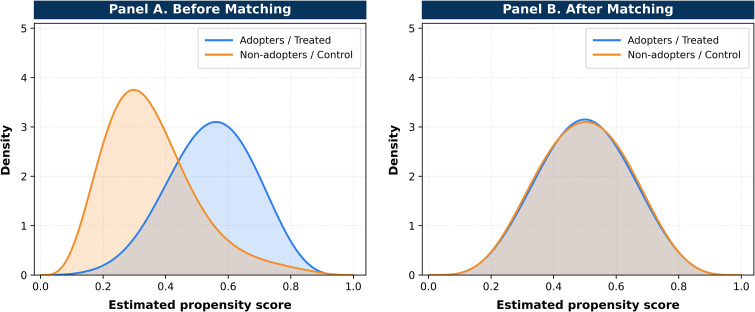
Propensity score distribution before and after matching for adopters and non-adopters.

The Fig 5 illustrates the distribution of the estimated propensity scores for adopters and non-adopters before and after matching. There are clear differences in the distribution of the adoption probabilities between the two groups, suggesting selection bias. Post-matching, the distributions are more similar, indicating better matching of the treated and control observations.

**Fig 6:**
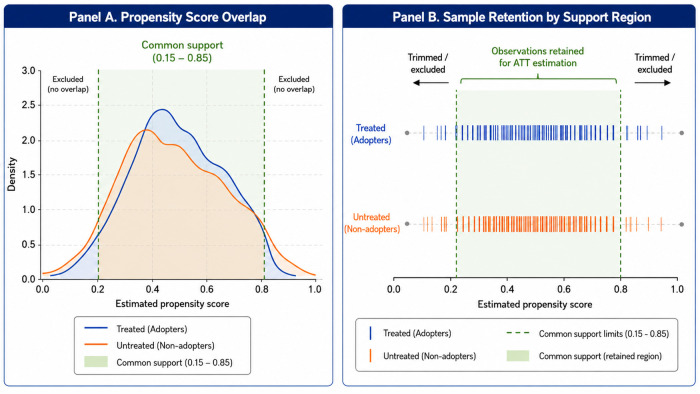
Common support region for treated and untreated observations.

The common support graph in Fig 6 illustrates the overlap of propensity scores between the adopters and non-adopters. The ATT was estimated using observations in the common support region. The adequate overlap ensures that adopters were matched with similar non-adopters, enhancing the validity of the estimated treatment effects.

### Perceptions and attitudinal structure

Table 11 summarizes farmers’ perceptions regarding the profitability, risk, cost-effectiveness, and institutional support associated with Sustainable Farming Practices (SFPs). The results indicate that 35% of farmers perceive SFPs as more profitable than conventional methods, while 46% believe profitability is about the same, and only 19.1% consider them less profitable. This suggests that the majority of farmers view SFPs as economically viable or at least comparable to traditional practices [30–33]. In terms of perceived risk, nearly half of the respondents (48.1%) identified a moderate risk, whereas only 21.9% felt the risk was high, implying that while some uncertainty remains, most farmers are cautiously optimistic about SFP outcomes.

**Table 11:** Farmers’ perceptions of profitability, risk, and cost of SFPs (N = 283).

Construct	Category	n	%
**Profitability vs. conventional**	More profitable	99	35.0
	About the same	130	46.0
	Less profitable	54	19.1
**Perceived risk level**	High	62	21.9
	Moderate	136	48.1
	Low	73	25.8
	None	12	4.2
**Cost-effectiveness judgment**	Cost-effective	161	56.9
	Costly	122	43.1
**Confidence to adopt SFPs**	Very confident	71	25.1
	Somewhat confident	139	49.1
	Not very confident	57	20.1
	Not confident at all	16	5.7
**Government support (perceived)**	Adequate	51	18.0
	Inadequate	232	82.0

Regarding cost perceptions, a majority (56.9%) found SFPs cost-effective, reflecting improved resource efficiency and reduced dependence on expensive chemical inputs. Confidence levels were generally positive, with nearly 74% of farmers being somewhat or very confident in their ability to adopt SFPs, indicating growing familiarity and self-assurance in using sustainable techniques. However, an overwhelming 82% of farmers reported inadequate government support, which highlights a critical institutional gap in facilitating widespread adoption [59]. Overall, these findings reveal that while farmers hold favorable economic and attitudinal perceptions toward SFPs, stronger government involvement through training, subsidies, and extension programs is essential to reduce risk perception and sustain long-term adoption. Farmers’ perceptions of the profitability, risk, and cost of adopting SFPs are presented in S4 Table in S2 File.

Table 12 presents the comparison of farmers’ perceived risk levels associated with sustainable farming practices (SFPs) across different education groups. The one-way ANOVA test reveals a significant difference in perceived risk among education levels (F(3, 279) = 18.2, p < 0.001), indicating that farmers’ educational attainment strongly influences how they perceive the risks of adopting sustainable practices. Farmers with no formal education reported the highest mean risk perception (3.8 ± 0.9), followed by those with primary education (3.4 ± 1.0). In contrast, farmers with secondary (2.9 ± 0.8) and tertiary education (2.6 ± 0.7) perceived significantly lower levels of risk [61].

**Table 12:** Comparison of perceived risk levels of SFPs across farmer education groups (ANOVA) (N = 283).

Education Level	n	Mean Risk Score (1 = Low, 5 = High)	Std. Dev.
**No formal**	50	3.8	0.9
**Primary**	74	3.4	1.0
**Secondary**	91	2.9	0.8
**Tertiary**	68	2.6	0.7
**Total (N = 283)**	283	3.1	1.0

ANOVA results: F(3, 279) = 18.2, p < 0.001*.

This pattern demonstrates that higher education is associated with lower perceived risk, likely due to better understanding of agronomic principles, greater exposure to innovation, and improved access to credible information sources. Educated farmers may also be more confident in experimenting with new techniques and evaluating outcomes objectively. Conversely, less educated farmers may rely on traditional knowledge and be more cautious toward unfamiliar practices. Overall, the results confirm that education plays a vital role in shaping farmers’ risk perceptions and adoption behavior, emphasizing the importance of investing in farmer education, awareness campaigns, and practical training programs to reduce psychological barriers to sustainable agriculture.

**Fig 7:**
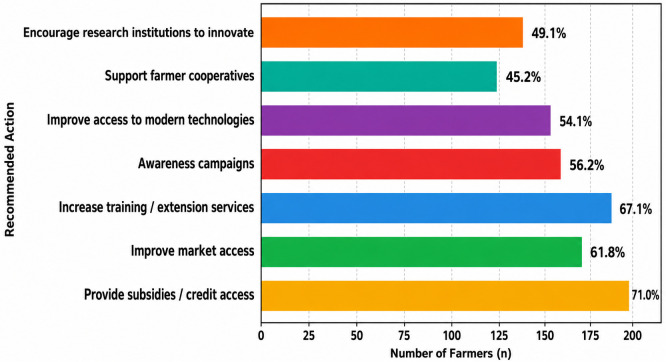
Farmers’ recommendations for government action (Multiple Response; N = 283).

Fig 7 summarizes farmers’ recommendations for government interventions to enhance the adoption of SFPs. The results reveal that respondents emphasized multiple areas where policy and institutional support are critically needed. The most frequently cited recommendation was to provide subsidies or improve access to agricultural credit (71.0%), highlighting the persistent financial barriers farmers face in transitioning toward sustainable practices. This was followed by calls to increase training and extension services (67.1%) and improve market access (61.8%), which reflect farmers’ demand for better knowledge dissemination, technical guidance, and fair market opportunities for sustainably produced crops. More than half of the respondents also stressed the importance of awareness campaigns (56.2%) and access to modern technologies (54.1%), underlining the need for government-led education and innovation programs to enhance the technical efficiency of farming systems [9]. Additionally, 49.1% encouraged greater involvement of research institutions, and 45.2% suggested supporting farmer cooperatives, which indicates a desire for stronger linkages between farmers, researchers, and extension networks.

Overall, these findings reveal that farmers view government support as a vital enabler of sustainable agricultural transformation. Policy measures that combine financial assistance, capacity building, technology transfer, and institutional coordination could substantially accelerate the adoption of SFPs and ensure that sustainability becomes a mainstream component of agricultural policy and rural development strategy in Pakistan.

Table 13 presents the results of an exploratory factor analysis (EFA) conducted to identify the underlying dimensions influencing farmers’ perceptions and attitudes toward sustainable farming practices. Using Principal Component Analysis with Varimax rotation, three distinct factors with eigenvalues greater than 1 were extracted, collectively explaining 68.4% of the total variance, an indication of a strong and reliable factor structure.

**Table 13:** Factor analysis of farmers’ perceptions and attitudes toward SFPs (Exploratory Factor Analysis; N = 283).

Item (Survey Question)	Factor 1: Economic Benefits	Factor 2: Environmental Concern	Factor 3: Institutional Support
**SFPs improve profitability**	0.78	0.21	0.15
**SFPs reduce production risk**	0.71	0.19	0.18
**SFPs improve soil fertility**	0.25	0.81	0.11
**SFPs mitigate climate change**	0.18	0.76	0.22
**Government should provide subsidies**	0.16	0.22	0.83
**Extension services important**	0.12	0.25	0.79
**Cooperatives can promote adoption**	0.19	0.28	0.74

*Extraction method: Principal Component Analysis (Varimax rotation).*

• *Eigenv*alues > 1 retained.

• Total va*riance explained = 68.4%.*

The first factor, labeled “Economic Benefits,” captures farmers’ belief that SFPs enhance profitability (loading = 0.78) and reduce production risk (loading = 0.71). This factor reflects the economic rationale behind adoption decisions, where perceived financial gains and stability motivate farmers to shift toward sustainable methods. The second factor, “Environmental Concern,” encompasses perceptions related to improved soil fertility (loading = 0.81) and climate change mitigation (loading = 0.76), highlighting the farmers’ awareness of ecological benefits and their role in promoting environmental stewardship. The third factor, “Institutional Support,” includes strong loadings for items such as government subsidies (0.83), extension services (0.79), and cooperative support (0.74), signifying the importance of external assistance and organized institutional frameworks in enabling adoption. The environmental perceptions of farmers regarding SFPs and concerns about climate change impacts are outlined in S5 and S6 Tables in S2 File.

Overall, the analysis reveals that farmers’ attitudes toward SFPs are shaped by a combination of economic, environmental, and institutional factors, suggesting that adoption is not solely profit-driven but also influenced by ecological values and policy support structures. Strengthening institutional mechanisms and linking financial incentives with environmental awareness could therefore enhance the long-term sustainability and widespread acceptance of SFPs.

### Farmer segmentation and pathway models

Table 14 presents the results of a cluster analysis that classifies farmers into distinct groups based on their adoption characteristics of Sustainable Farming Practices (SFPs). The analysis identified three major clusters, each representing a unique behavioral and socioeconomic profile.

**Table 14:** Cluster analysis of farmer groups based on adoption characteristics (N = 283).

Cluster	Key Features	n	%
**Cluster 1: Progressive adopters**	Educated, larger farms (>3 acres), access to training & credit, high profitability	96	33.9
**Cluster 2: Cautious adopters**	Moderate education, small–medium farms, partial adoption, concerned about costs	79	27.9
**Cluster 3: Traditional non-adopters**	Low education, small farms (<2 acres), no training, high risk perception	108	38.2
**Total**		**283**	**100.0**

Cluster 1 describes that progressive adopters (33.9%) consist of well-educated farmers with larger landholdings (over 3 acres) who have access to training programs and agricultural credit. These farmers demonstrate high profitability, strong technical awareness, and positive attitudes toward innovation, positioning them as leaders in sustainability transition. Cautious adopters (27.9%) include farmers with moderate education levels and small to medium-sized farms in Cluster 2. They have partially adopted SFPs but remain hesitant due to concerns over costs, risks, and uncertain short-term returns. This group represents the segment most likely to benefit from targeted interventions such as training, credit facilitation, and market assurance mechanisms. Cluster 3 presents that traditional non-adopters (38.2%) are characterized by low education levels, small landholdings (below 2 acres), lack of training, and high-risk perception. These farmers are typically resource-constrained and rely heavily on conventional practices, making them the most vulnerable to environmental and economic challenges.

Overall, the cluster analysis illustrates the heterogeneity among farmers regarding awareness, resources, and willingness to adopt SFPs. Policy measures should therefore be differentiated and cluster-specific—encouraging progressive adopters to act as sustainability champions, supporting cautious adopters through financial and technical assistance, and prioritizing training and outreach programs for traditional non-adopters to build confidence and reduce perceived risks. The scree plot of factor analysis is displayed in S4 Fig in S1 File.

Table 15 summarizes the results of the SEM conducted to evaluate both the direct and indirect relationships among institutional support, environmental concern, adoption of SFPs, and farm profitability. The model demonstrates strong statistical validity and excellent fit indices (χ²/df = 2.1; RMSEA = 0.048; CFI = 0.96; TLI = 0.95), indicating that the hypothesized relationships are well supported by the data.

**Table 15:** Structural Equation Modeling (SEM) results: path coefficients and model fit.

Path	Std. Coefficient (β)	t-value	p-value
**Institutional support → Adoption**	0.42	6.12	<0.001***
**Environmental concern → Adoption**	0.27	4.28	<0.001***
**Adoption → Profitability**	0.49	7.01	<0.001***
**Direct path: Institutional support → Profitability**	0.18	2.76	0.006**

***Hypothesized Model:***

• *Institutional Support → Adoption → Profitability*

• *Environmental Concern → Adoption → Profitability*

*Model fit indices:*

*• χ²/df = 2.1 (acceptable ≤ 3.0)*

• *RMSEA = 0.048 (good < 0.05)*

• *CFI = 0.96 (good ≥ 0.95)*

• *TLI = 0.95 (good ≥ 0.95)*

The path coefficients reveal that institutional support (β = 0.42, t = 6.12, p < 0.001) and environmental concern (β = 0.27, t = 4.28, p < 0.001) both exert significant positive effects on the adoption of SFPs, suggesting that farmers are more likely to adopt sustainable practices when supported by government programs, extension services, and cooperatives, as well as when they are aware of environmental challenges. Furthermore, the adoption of SFPs has a strong positive influence on profitability (β = 0.49, t = 7.01, p < 0.001), confirming that sustainability-oriented practices not only enhance environmental outcomes but also improve economic performance [63–65]. Additionally, the direct effect of institutional support on profitability (β = 0.18, t = 2.76, p = 0.006) remains significant even after accounting for adoption, indicating a partial mediation effect, where institutional support contributes both directly and indirectly to improving farm profitability through increased adoption.

Overall, the SEM results provide compelling evidence that institutional support and environmental awareness jointly drive sustainable adoption behavior, which in turn leads to higher profitability. These findings underscore the importance of integrated policy frameworks that combine environmental education, institutional assistance, and incentive structures to ensure a synergistic and sustained transition toward environmentally and economically resilient agriculture.

To our knowledge, this is the first systematic analysis that quantifies both the determinants and the economic returns of adopting SFPs in KPK rice systems, thereby providing robust evidence to guide future policy and extension efforts.

## Discussion

Overall, the findings show that sustainable farming practice (SFP) adoption in KPK rice systems is shaped by a combination of human capital, farm resources, institutional support, and market proximity. Across models, training exposure, access to credit/subsidy, education, and farm size consistently increase the likelihood of adoption, while remoteness and higher transaction costs reduce it. Importantly, the economic results indicate that adoption is not only environmentally relevant but also financially attractive under farmer conditions, supporting the argument that sustainability and profitability can be aligned in smallholder rice systems.

### Determinants of sustainable farming adoption

The results indicate that adoption of SFPs in Khyber Pakhtunkhwa is shaped primarily by human capital, financial access, institutional support, and market connectivity [4,5,7,9]. Education consistently emerges as a significant determinant, suggesting that better-educated farmers are more capable of processing technical information, assessing risk, and experimenting with innovative practices [45]. This aligns with diffusion theory [66], which emphasizes the role of knowledge and cognitive capacity in accelerating technology uptake. Training exposure appears as the strongest predictor of adoption across models. Farmers who received formal training were substantially more likely to adopt SFPs, highlighting the importance of extension services and experiential learning [42]. In smallholder systems like those in KPK, many sustainable practices require adjustments in input management, water scheduling, and soil treatment. Without hands-on guidance, farmers may hesitate to transition from conventional systems [7].

Access to credit or subsidies also significantly increases adoption likelihood. This confirms that liquidity constraints remain a major barrier, particularly for smallholders who face upfront transition costs and uncertainty regarding short-term returns [26]. The positive role of farm size further suggests that resource endowment influences risk-bearing capacity and investment potential. Conversely, distance to market negatively affects adoption [36,53]. Farmers located farther from markets may face higher transaction costs, weaker access to information, and reduced incentives to produce sustainably if premium markets are inaccessible [42]. This finding underscores the importance of rural infrastructure and market integration in sustainability transitions. Farmers’ confidence in adopting SFPs and willingness to recommend SFPs to others are summarized in S7 and S8 Tables in S2 File. Further, the questionnaire used while surveying is provided in S9 Table in S2 File.

Together, these results demonstrate that SFP adoption in KPK is not purely a matter of environmental awareness; it depends on an enabling ecosystem combining knowledge, finance, and institutional access.

### Economic implications of sustainable farming

The economic analysis provides strong evidence that SFP adoption is financially viable. Cost–benefit comparisons reveal that sustainable systems generate higher net income and stronger benefit–cost ratios than conventional practices [38]. Importantly, this advantage is driven not merely by cost reduction but by improved yield performance, better resource efficiency, and modest price advantages [57].

The regression and propensity score matching results reinforce these findings by showing that adopters achieve significantly higher profitability, yield, and household income even after controlling for observable characteristics. This strengthens confidence that the economic gains are associated with adoption rather than purely farmer heterogeneity [10].

These findings challenge the common perception that sustainable agriculture sacrifices productivity for environmental benefits. In the KPK rice system, sustainability appears to enhance both environmental performance and economic returns. This dual benefit is particularly important for smallholders, for whom profitability remains the primary decision criterion. While the cross-sectional nature of the data limits full causal inference, the combination of matching, regression adjustment, and sensitivity analysis increases confidence in the robustness of the estimated economic effects.

### Perceptions, risk, and behavioral dimensions

Farmers generally perceive SFPs as profitable and environmentally beneficial [14]. However, risk perception varies significantly across education levels, indicating that psychological and informational barriers still influence decision-making [15,53].

The factor analysis reveals three core dimensions shaping farmer attitudes: economic benefits, environmental concern, and institutional support [26]. This suggests that adoption decisions are multidimensional. Farmers are motivated not only by profit expectations but also by ecological awareness and confidence in institutional backing [56].

Cluster analysis further highlights heterogeneity among farmers. Progressive adopters possess education, credit access, and training exposure, while traditional non-adopters are characterized by limited resources and higher perceived risk. This segmentation indicates that a uniform policy approach may be ineffective; differentiated strategies are required.

### Institutional support and structural constraints

The findings consistently show that institutional support plays a critical role in shaping adoption and profitability [40,42]. Structural equation modeling confirms that institutional support influences adoption directly and indirectly through improved sustainability practices [44]. At the same time, farmers report significant constraints, including transition costs, limited credit access, climate variability, and weak market incentives [46]. These structural barriers explain why adoption remains incomplete despite favorable perceptions [62]. Thus, adoption should be understood as a systemic process requiring coordination between farmers, extension services, credit institutions, and markets.

### Linking findings to the KPK context

The specific context of Khyber Pakhtunkhwa is important. Rice farmers in this region are predominantly small-scale, resource-constrained, and dependent on local markets. Limited cooperative participation and uneven extension coverage amplify structural barriers [62]. Given these conditions, sustainability transitions in KPK require stronger decentralized extension systems, targeted financial mechanisms for smallholders, improved rural infrastructure, and market development for sustainably produced rice. The evidence suggests that when these enabling factors are present, adoption increases substantially and leads to improved economic outcomes.

### Practical implications

The results provide clear guidance for farmers and extension practitioners. Training programs and demonstration plots should be expanded to reduce uncertainty and improve technical competence. Peer-learning networks can leverage progressive adopters as local champions to influence cautious and traditional farmers. Facilitating affordable credit mechanisms can reduce transition barriers. Market linkages should be strengthened through aggregation centers and cooperative marketing models, particularly in remote areas. Importantly, communicating clear cost–benefit evidence can help overcome skepticism and encourage informed decision-making.

### Policy implications

The findings from this study provide several key insights for enhancing the adoption of sustainable farming practices among rice farmers in Khyber Pakhtunkhwa, Pakistan. The following policy implications are grounded in the results and discussion of the manuscript, and each recommendation is accompanied by its justification and potential modes of implementation:

#### Expand access to credit and subsidies for smallholder farmers.

Access to credit and subsidies significantly increases the likelihood of adopting SFPs, as demonstrated by the logistic regression results (β = 0.951, p < 0.001), which show that farmers with credit access are 2.6 times more likely to adopt sustainable practices. Additionally, the PSM results highlight that adopters of SFPs achieve higher profitability, yield, and household income compared to non-adopters, with financial access playing a critical role in this improvement. Government and financial institutions should introduce low-interest loans and flexible repayment terms targeted specifically at smallholders who wish to invest in sustainable farming equipment and practices. Local agricultural banks or microfinance institutions could collaborate with extension services to create targeted financial products that align with the cash flow of smallholder farmers. Training programs should include financial literacy components to help farmers manage these financial resources effectively.

#### Strengthen agricultural extension services with focus on SFPs.

Training exposure was found to be the strongest predictor of SFP adoption, with trained farmers being 3.5 times more likely to adopt sustainable practices (logistic regression result: β = 1.261, p < 0.001). However, the results also indicate that a significant gap remains, as 58% of farmers had not received any form of training on SFPs. Expanding training programs is crucial for converting awareness into sustained adoption. The provincial agricultural department should increase investment in extension services, ensuring that they reach rural and remote areas with tailored training on SFPs. Farmer Field Schools (FFS) and demonstration plots should be established to provide hands-on learning experiences for farmers, showcasing the long-term benefits and profitability of adopting SFPs [67]. Partnerships with universities and research institutions can bring in new knowledge and practical solutions, which can be directly transferred to farmers through localized workshops and extension services.

#### Improve market access for sustainably produced rice.

Proximity to markets was found to have a significant positive impact on SFP adoption, with farmers located closer to markets being more likely to adopt these practices (χ² = 11.5, p < 0.01). Additionally, farmers who perceive their practices as more profitable tend to adopt SFPs, and better market access is linked to higher prices for sustainably produced rice (mean price of PKR 80/kg for sustainable rice). This indicates that improving market access can incentivize wider adoption of SFPs by offering better returns for sustainably produced crops.

Policies should focus on improving rural infrastructure, including roads, transportation, and market facilities, to reduce the cost and time associated with getting products to market. Government and private sector partnerships could facilitate the creation of agricultural cooperatives that help farmers gain better bargaining power and access to niche markets for sustainable rice. Digital platforms could be developed to connect farmers with consumers or wholesalers, ensuring a steady demand for sustainably produced rice.

#### Provide incentives for long-term adoption of SFPs.

The study reveals that although more than half of farmers have adopted at least one SFP, the duration of adoption is relatively short, with 39.6% of adopters implementing these practices within the last two years. The short-term adoption pattern suggests that while farmers are open to SFPs, consistent support is needed to maintain long-term adoption. This is further corroborated by the findings indicating that transition costs and yield risks remain significant barriers.

Implement performance-based incentives that reward farmers for continued use of sustainable practices over time, such as subsidies for maintaining soil health, water management, or organic farming practices. Establish a reward system for farmers who demonstrate measurable improvements in sustainability, such as reductions in water use or chemical inputs, with certification or labeling schemes that enhance the marketability of their products. Long-term monitoring and advisory support can ensure that farmers are able to mitigate risks associated with transitioning to and maintaining SFPs, such as yield uncertainty during the adoption phase.

## Conclusion

This study examines the factors influencing the adoption of sustainable farming practices among rice farmers in Khyber Pakhtunkhwa, Pakistan. The results indicate that adoption is driven by factors such as education, farm size, access to credit, training exposure, and market proximity. Farmers with higher education, larger farms, and access to training were more likely to adopt sustainable practices, which in turn led to higher yields and greater profitability. This demonstrates that SFP adoption can enhance both environmental sustainability and farm profitability. However, several barriers to adoption remain, including high transition costs, limited access to credit, climate variability, and insufficient training. These constraints need to be addressed through targeted policies that support farmers in overcoming financial and informational challenges.

While the study provides valuable insights, it also has limitations. The cross-sectional data used in this research limits causal inference, as unobserved factors may still influence adoption decisions. Future studies could use longitudinal data to better understand the long-term impacts of SFP adoption. Additionally, the focus on household-level adoption without considering plot-specific factors may limit the precision of the findings. Incorporating plot-level data would provide a more detailed understanding of adoption patterns across different land types. Furthermore, the study was conducted only in KPK's rice-growing districts, so findings may not fully apply to other regions or farming systems. Multi-province studies could strengthen the external validity of the results.

Future research should focus on tracking SFP adoption over time through longitudinal studies, which would provide insights into the sustainability and long-term benefits of these practices. Investigating the role of social networks and farmer-to-farmer learning could also provide valuable information about the adoption process. Additionally, incorporating biophysical data, such as soil health and water use efficiency, would offer a more comprehensive understanding of the environmental benefits of SFPs. Research focusing on specific farmer groups, such as those with lower education levels or smaller landholdings, would help develop targeted interventions to address their unique challenges.

The findings of this study have important policy implications. Expanding access to credit and subsidies for smallholder farmers is crucial, as financial constraints were identified as a major barrier to adoption. Providing affordable credit and tailored financial products would help smallholders invest in sustainable farming technologies. Strengthening agricultural extension services and offering more training opportunities, particularly in rural areas, is also essential for ensuring farmers have the technical knowledge to adopt SFPs. Improving market access for sustainably produced rice could provide farmers with the financial incentive to adopt sustainable practices. Policies focused on improving rural infrastructure and market facilities would reduce transaction costs and make it easier for farmers to access markets. Finally, providing long-term incentives for SFP adoption, such as performance-based rewards or certifications, would encourage farmers to maintain sustainable practices over time.

Overall, while adoption of SFPs in KPK can improve environmental and economic outcomes, overcoming barriers such as financial constraints and limited training is essential for broader implementation. Targeted policy interventions can support farmers in adopting and maintaining sustainable practices, contributing to both environmental protection and enhanced livelihoods.

## Supporting information

S1 File Supporting Information Figures. (PDF)

S2 File Supporting Information Tables. (PDF)
