# Supplementary material for: Factors influencing the adoption of sustainable rice farming practices in Khyber Pakhtunkhwa, Pakistan
Source: PLoS One. 2026 Jun 9;21(6):e0350735. doi: 10.1371/journal.pone.0350735 (PMC13249190; doi:10.1371/journal.pone.0350735)
Supplement: S1 File — (PDF) [file pone.0350735.s001.pdf]

Adoption Intensity and Index of Sustainable Farming Practices (SFPs)

This section provides a detailed explanation of the construction, estimation, and interpretation of the Sustainable Farming Practices (SFP) adoption intensity and index measures. It addresses reviewer concerns regarding the potential oversimplification of adoption as a binary variable.

While the main manuscript models adoption as a binary decision (adopter vs non-adopter), farmers may adopt multiple sustainable practices simultaneously. To capture this behavioral complexity, an adoption intensity measure and a normalized adoption index were constructed using practice-level data.

Eight sustainable farming practices were included in the dataset:

1) Crop rotation/intercropping
2) Reduced chemical/pesticide use
3) Water-saving methods (e.g., AWD)
4) Natural fertilizers/compost
5) Soil testing
6) Biological pest control
7) Organic practices (certified/in transition)
8) Cover crops/green manuring

Each practice was coded as:

$$SFP_{ij} = \begin{cases} 1 & \text{if farmer } i \text{ adopted practice } j \\ 0 & \text{otherwise} \end{cases}$$

Adoption Intensity (Count Measure):

$$\text{SFP\_Count}_i = \sum_{j=1}^{8} SFP_{ij}$$

This measure captures the number of sustainable practices adopted by farmer i.
Theoretical range: 0–8.
Observed range in sample: 0–6.

Adoption Index (Normalized Measure):

$$\text{SFP\_Index}_i = \frac{\text{SFP\_Count}_i}{8}$$

This index scales adoption between 0 and 1 for comparability across contexts.

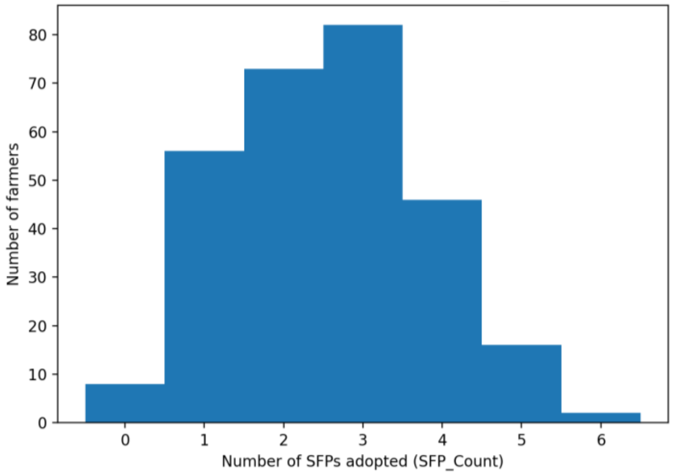

S1 Fig. Distribution of adoption intensity

The histogram illustrates that most farmers adopt between two and three practices, with very few adopting none or more than five.

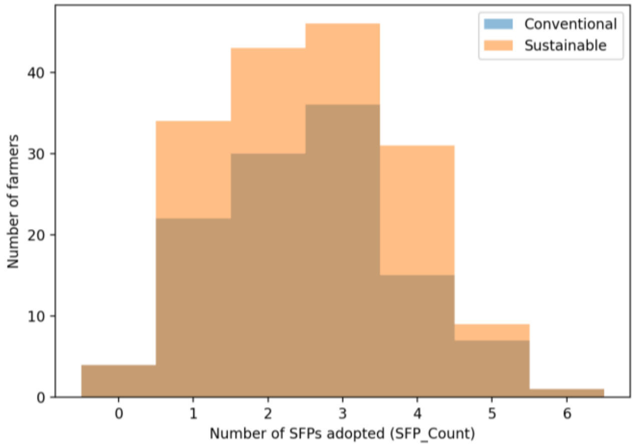

S2 Fig. Adoption intensity by farming system group

The boxplot comparison suggests overlapping intensity distributions across farming system groups, indicating heterogeneity within both categories.

Because SFP_Count is a non-negative integer variable, a Poisson regression model was estimated:

$$E(\text{SFP\_Count}_i \mid X_i) = \exp\,(\beta_0 + \beta X_i)$$

Where:

- X_i includes education, farm size, credit access, training exposure, market distance, and perceived profitability.
- Results are reported as Incidence Rate Ratios (IRR):

- IRR > 1 → higher adoption intensity
- IRR < 1 → lower adoption intensity

Results are reported as Incidence Rate Ratios (IRR). An IRR greater than 1 implies higher adoption intensity, while an IRR less than 1 implies lower intensity.

The intensity regression indicates that while farmers adopt multiple practices, the key socioeconomic and institutional variables identified in the binary model do not significantly explain variation in the number of practices adopted. This suggests that adoption intensity may depend on practice-specific constraints, local input availability, or unobserved farmer preferences. Meanwhile, perceived profitability (9%) and distance to market (5%) had moderate impacts. While profitability perceptions influence farmers' motivation to adopt, greater market distance tends to discourage adoption by increasing transaction costs and limiting input access. Overall, the decision tree analysis confirms that human capacity, financial empowerment, and farm resources are the most decisive predictors of SFP adoption. These findings advocate for integrated policy strategies—combining training, credit facilitation, and market support—to effectively enhance the diffusion of sustainable farming across diverse farmer groups. Decision three variable importance for adoption is illustrated in S3 Fig.

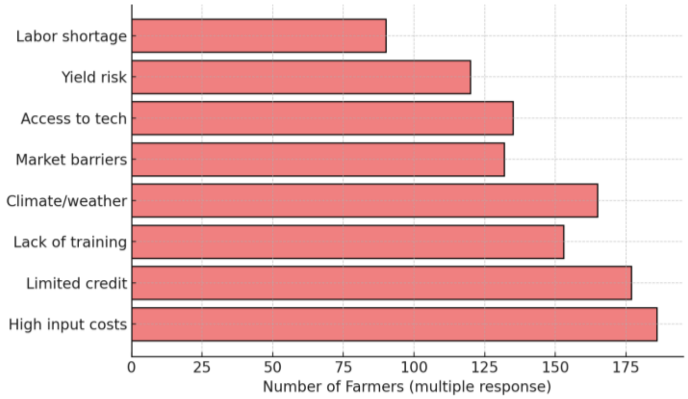

S3 Fig. Challenges faced in the adoption of SFPs

The Scree plot of factor analysis is displayed in S4 Fig. The cluster analysis illustrates the heterogeneity among farmers regarding awareness, resources, and willingness to adopt SFPs. Policy measures should therefore be differentiated and cluster-specific—encouraging progressive adopters to act as sustainability champions, supporting cautious adopters through financial and technical assistance, and prioritizing training and outreach programs for traditional non-adopters to build confidence and reduce perceived risks.

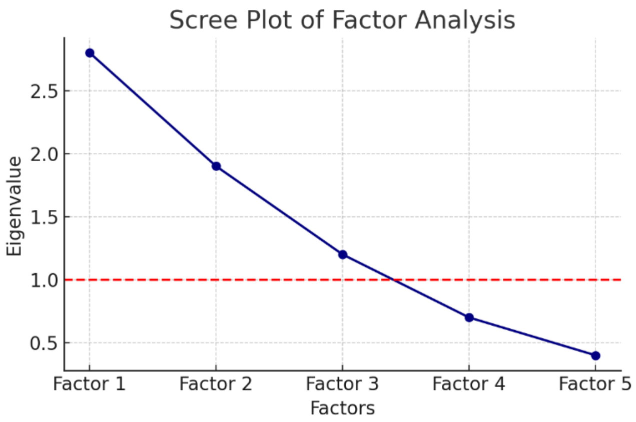

S4 Fig. Scree plot of factor analysis

Notably, our findings resonate with global research on SFP adoption, suggesting that many drivers and constraints are universal rather than region-specific. For instance, the importance of farmer education, access to credit, and extension contact in boosting SFP uptake mirrors patterns observed in other rice-growing countries. Extensive studies in South Asia similarly report that better-informed and resourced farmers are more likely to adopt climate-smart practices, whereas a lack of awareness and financial capacity hinders others. The barriers identified in KPK, such as limited technical support or the costs of new equipment, also echo those faced by smallholders in Southeast Asia and Africa. Even in technologically advanced rice economies like Vietnam, farmers grapple with sustainability challenges, including over-reliance on chemicals, high input costs, and climate risks. That both well-equipped and resource-constrained farming communities struggle with adopting SFPs underscores a key insight: common challenges cut across diverse contexts, reinforcing the broader relevance of our study's results to global sustainable agriculture efforts.
