# Supplementary material for: Factors influencing the adoption of sustainable rice farming practices in Khyber Pakhtunkhwa, Pakistan
Source: PLoS One. 2026 Jun 9;21(6):e0350735. doi: 10.1371/journal.pone.0350735 (PMC13249190; doi:10.1371/journal.pone.0350735)
Supplement: S2 File — (PDF) [file pone.0350735.s002.pdf]

Adoption Intensity and Index of Sustainable Farming Practices (SFPs)

Eight sustainable farming practices were included in the dataset:

1) Crop rotation/intercropping
2) Reduced chemical/pesticide use
3) Water-saving methods (e.g., AWD)
4) Natural fertilizers/compost
5) Soil testing
6) Biological pest control
7) Organic practices (certified/in transition)
8) Cover crops/green manuring

Each practice was coded as:

$$SFP_{ij} = \begin{cases} 1 & \text{if farmer } i \text{ adopted practice } j \\ 0 & \text{otherwise} \end{cases}$$

Adoption Intensity (Count Measure):

$$\text{SFP\_Count}_i = \sum_{j=1}^{8} SFP_{ij}$$

This measure captures the number of sustainable practices adopted by farmer i.
Theoretical range: 0–8.
Observed range in sample: 0–6.

Adoption Index (Normalized Measure):

$$\text{SFP\_Index}_i = \frac{\text{SFP\_Count}_i}{8}$$

This index scales adoption between 0 and 1 for comparability across contexts.

S1 Table. Descriptive Statistics of Adoption Intensity

**Statistic**	**Value**
**Mean SFP_Count**	2.56
**Standard Deviation**	1.25
**Median**	3
**Minimum**	0

**Maximum Observed**	6
**Mean SFP_Index**	0.320

The results show that farmers adopt on average 2–3 sustainable practices. This confirms that adoption behavior is multi-dimensional rather than purely binary.

Because SFP_Count is a non-negative integer variable, a Poisson regression model was estimated:

$$E(\text{SFP\_Count}_i \mid X_i) = \exp\,(\beta_0 + \beta X_i)$$

Where:

- X_i includes education, farm size, credit access, training exposure, market distance, and perceived profitability.
- Results are reported as Incidence Rate Ratios (IRR):
  - IRR > 1 → higher adoption intensity
  - IRR < 1 → lower adoption intensity

Results are reported as Incidence Rate Ratios (IRR). An IRR greater than 1 implies higher adoption intensity, while an IRR less than 1 implies lower intensity.

The intensity regression indicates that while farmers adopt multiple practices, the key socioeconomic and institutional variables identified in the binary model do not significantly explain variation in the number of practices adopted. This suggests that adoption intensity may depend on practice-specific constraints, local input availability, or unobserved farmer preferences.

S2 Table. Poisson Regression

Variable	IRR	p-value
**Education (ordinal)**	1.020	0.773
**Farm size (acres)**	0.928	0.234
**Credit access (Yes=1)**	1.008	0.912
**Training (Yes=1)**	1.079	0.315
**Market distance (km)**	1.013	0.377
**Perceived profitability**	0.991	0.859

The supplementary intensity analysis strengthens the manuscript by demonstrating that adoption is multi-layered. While system-level transition (binary adoption) is strongly driven by training, credit access, and perceived profitability, the depth of adoption appears more heterogeneous. This supports the main conclusions without altering the core findings.

S3 Table. Variable Importance Ranking from Decision Tree Model for Predicting SFP Adoption

Rank	Predictor Variable	Importance (%)
**1**	Training received	32.0
**2**	Access to credit/subsidy	24.0
**3**	Education level	18.0
**4**	Farm size	12.0
**5**	Perceived profitability	9.0
**6**	Distance to market	5.0

Model accuracy = 78.5% (10-fold cross-validation).

S3 Table presents the results of a Decision Tree model used to identify and rank the most influential predictors of farmers' adoption of Sustainable Farming Practices (SFPs). The model achieved a high classification accuracy of 78.5% through 10-fold cross-validation, indicating strong predictive reliability. Among the variables included, training received (importance = 32%) emerged as the most powerful determinant of adoption. This underscores the critical role of knowledge dissemination, technical exposure, and capacity-building programs in influencing farmers' willingness and ability to implement sustainable techniques [26-29]. The second most influential factor was access to credit or subsidies (24%), highlighting the importance of financial accessibility in facilitating the transition to sustainable farming systems. Education level (18%) ranked third, suggesting that farmers with greater literacy and comprehension skills are more open to innovation and environmental responsibility. Farm size (12%) also contributed significantly, implying that larger farms have more flexibility and resources to experiment with sustainable inputs and technologies.

S4 Table summarizes farmers' perceptions regarding the profitability of Sustainable Farming Practices (SFPs) compared to conventional farming methods. The results indicate that a majority of farmers view SFPs as economically beneficial, with 32.2% perceiving them as more than 10% more profitable, and 24.0% considering them slightly more profitable. Together, these groups account for over half of the respondents (56.2%), demonstrating a widespread belief that sustainable practices contribute to improved economic returns. In contrast, 27.9% of farmers perceived no major difference in profitability, while only 15.9% regarded SFPs as less profitable.

S4 Table. Farmers' Perceptions of Profitability of SFPs Compared to Conventional Practices (N = 283)

Perception	n	%
**More profitable (>10% higher)**	91	32.2
**Slightly more profitable (1–10% higher)**	68	24.0
**About the same**	79	27.9
**Less profitable**	45	15.9
**Total**	**283**	**100.0**

These findings reveal a generally positive economic perception of sustainability among farmers, which aligns with earlier analyses showing higher yields and net returns for adopters. The results suggest that farmers increasingly recognize the long-term financial viability of SFPs through reduced input dependency, improved soil productivity, and resilience against environmental stress. However, the presence of a notable minority who still perceive SFPs as less profitable reflects uncertainty during the transition period and possibly inadequate market incentives. Therefore, consistent government and institutional support—such as price premiums, input subsidies, and targeted awareness programs—is essential to further strengthen farmers' confidence in the profitability and sustainability of these practices.

S5 Table highlights farmers' perceptions regarding the environmental benefits of Sustainable Farming Practices (SFPs). The findings reveal that a large majority of respondents expressed positive views toward the ecological outcomes of adopting SFPs. Specifically, 39.9% strongly agreed and 33.9% agreed that sustainable farming practices are environment-friendly, collectively representing nearly three-fourths (73.8%) of the total sample. Meanwhile, 18.0% remained neutral, and only 8.1% disagreed, indicating minimal skepticism [22].

S5 Table. Farmers' Perceptions About Environmental Benefits of Sustainable Farming Practices (N = 283)

Perception	n	%
**Strongly agree that SFPs are environment-friendly**	113	39.9
**Agree**	96	33.9
**Neutral**	51	18.0
**Disagree**	23	8.1
**Total**	**283**	**100.0**

These results suggest a strong environmental awareness among farmers, reflecting growing recognition of the role of sustainable agriculture in reducing pollution, conserving soil fertility, and mitigating climate change effects. The findings also imply that awareness campaigns, extension activities, and peer learning may have successfully enhanced farmers' understanding of the environmental significance of sustainable methods. However, the presence of neutral and dissenting respondents shows that continued efforts are needed to strengthen environmental

education and evidence-based demonstration programs. Overall, the results affirm that most farmers perceive SFPs not merely as an economic choice but also as a responsible approach toward ecological sustainability and long-term agricultural resilience.

S6 Table presents farmers' levels of concern regarding the impacts of climate change on rice farming. The results indicate that a substantial majority of respondents view climate change as a serious threat to agricultural productivity and livelihoods. Specifically, 42.0% of farmers reported being very concerned, while 33.9% were somewhat concerned, together representing nearly three-fourths (75.9%) of the sample. In contrast, only 15.9% were not very concerned and 8.1% were not concerned at all, suggesting that a small minority remain less aware or less affected by climate-related challenges.

S6 Table. Farmers' Concern About Climate Change Impacts on Rice Farming (N = 283)

Concern Level	n	%
**Very concerned**	119	42.0
**Somewhat concerned**	96	33.9
**Not very concerned**	45	15.9
**Not concerned at all**	23	8.1
**Total**	283	100.0

These findings underscore that climate awareness among rice farmers is relatively high, reflecting their direct exposure to changing weather patterns, irregular rainfall, pest outbreaks, and temperature fluctuations that affect crop yields. The data also suggest that farmers' growing concern may act as a motivating factor for adopting climate-resilient and sustainable practices, such as water-saving techniques, soil conservation, and reduced chemical dependency. However, the presence of a small segment of unconcerned farmers highlights the need for targeted awareness programs and localized climate adaptation training to ensure that all farmers are adequately informed and prepared to mitigate climate-related risks. Overall, the results demonstrate that climate change is widely perceived as an urgent issue among rice farmers, influencing their attitudes toward sustainability and resilience in agriculture.

S7 Table describes the levels of confidence expressed by farmers regarding their ability to adopt Sustainable Farming Practices (SFPs). The results reveal that a majority of respondents felt reasonably confident in their capability to implement such practices. Specifically, 49.1% of farmers reported being somewhat confident, while 25.1% considered themselves very confident, together representing nearly three-fourths (74.2%) of the sample [6]. Conversely, 20.1% of farmers were not very confident, and only 5.7% expressed no confidence at all, indicating a relatively small portion of farmers who perceive significant barriers or lack the necessary skills.

S7 Table. Farmers' Confidence in Their Ability to Adopt Sustainable Farming Practices (N = 283)

Confidence Level	n	%
**Very confident**	71	25.1
**Somewhat confident**	139	49.1
**Not very confident**	57	20.1

**Not confident at all**	16	5.7
**Total**	283	100.0

These findings suggest that farmers generally possess a positive self-assessment of their technical and managerial abilities, likely due to growing exposure to extension programs, peer learning, and demonstration plots promoting sustainable agriculture. However, the presence of about one-fourth of farmers with low confidence levels points to a continued need for capacity-building interventions—including hands-on training, field demonstrations, and access to advisory services—to strengthen practical competence and reduce uncertainty. Overall, the results highlight a favorable psychological readiness among most farmers, which is crucial for the successful scaling-up of SFP adoption in the study region.

S8 Table presents farmers' willingness to recommend Sustainable Farming Practices (SFPs) to other members of the farming community. The data indicate a strong positive perception and acceptance of SFPs among respondents. A significant majority, 73.1%, expressed that they would recommend SFPs to fellow farmers, while only 26.9% stated they would not. This overwhelming inclination toward recommending sustainable methods reflects a high level of satisfaction and trust in the outcomes of these practices, including improved profitability, soil health, and long-term sustainability [19].

S8 Table. Willingness to Recommend Sustainable Farming Practices (SFPs) to Other Farmers (N = 283)

**Response**	**n**	**%**
**Yes**	207	73.1
**No**	76	26.9
**Total**	**283**	**100.0**

The findings suggest that farmers who have adopted or experienced the benefits of SFPs are likely to act as change agents within their local communities, promoting peer learning and accelerating the diffusion of sustainable technologies. The minority who was reluctant to recommend may be influenced by perceived uncertainties, higher initial costs, or insufficient institutional support. Therefore, strengthening farmer-to-farmer extension approaches, demonstration farms, and incentive programs could further enhance adoption momentum. Overall, the results highlight that social influence and experiential learning play a critical role in scaling up sustainability initiatives in agriculture.

Notably, the findings resonate with global research on SFP adoption, suggesting that many drivers and constraints are universal rather than region-specific. For instance, the importance of farmer education, access to credit, and extension contact in boosting SFP uptake mirrors patterns observed in other rice-growing countries. Extensive studies in South Asia similarly report that better-informed and resourced farmers are more likely to adopt climate-smart practices, whereas a lack of awareness and financial capacity hinders others. The barriers identified in KPK, such as limited technical support or the costs of new equipment, also echo those faced by smallholders in Southeast Asia and Africa. Even in technologically advanced rice economies like Vietnam, farmers grapple with sustainability challenges, including over-reliance on chemicals, high input costs, and climate risks. That both well-equipped and resource-constrained farming communities struggle

with adopting SFPs underscores a key insight: common challenges cut across diverse contexts, reinforcing the broader relevance of our study's results to global sustainable agriculture efforts.

S9 Table: Questionnaire

Demographic Information

Name: ________________________ Gender: ☐ Male ☐ Female

Area __________________________ Phone/Email: __________________________

Age: __________________________ Qualification: ____________________________

Working experience: ________Years Total Land Area Owned (Acres): ____________

Household Head: _______________ Household Size: __________________________

Section 1: Socioeconomic Factors

1. Do you belong to a farmer cooperative or association?

☐ Yes ☐ No

If yes specify please ____

2. How do you perceive the social pressure to adopt sustainable farming practices?

☐ High ☐ Moderate ☐ Low ☐ None

3. How do you perceive the support from your family and community for adopting sustainable farming practices?

☐ Very supportive ☐ Somewhat supportive

☐ Not very supportive ☐ Not supportive at all

4. What is your major source/s of income?

☐ Farming ☐ Non-farming

☐ Business ☐ Employment ☐ Other (specify: ______)

5. What is your annual income? _____________________________ (PKR)

6. Have you received any government subsidies or incentives for sustainable farming practices?

☐ Yes ☐ No

If yes, what types of subsidies or incentives have you received? (List types: ____)

7. Do you have access to extension services for sustainable farming?

☐ Yes ☐ No

If yes, what types of services or programs have you accessed? (List types: ____)

8. Do you have access to modern technologies for sustainable farming?

☐ Yes ☐ No

9. Do you have access to agricultural extension services for sustainable farming practices?

☐ Yes ☐ No

10. Do you have access to markets for selling sustainably produced rice?

☐ Yes ☐ No

11. How far is the nearest market from your farm?

☐ Less than 5 km ☐ 6-10 km

☐ 11-20 km ☐ More than 20 km

Section 2: Sustainable Farming Practices & Adoption

12. Do you know about sustainable farming practices for rice production?

☐ Yes ☐ No

13. How did you learn about sustainable farming practices?

☐ Extension department ☐ Research institutions

☐ Private companies ☐ NGOs

☐ Fellow farmers ☐ Progressive farmers

☐ Internet or social media ☐ Other (please specify)

____

14. Do you currently use any sustainable farming practice for rice production?

☐ Yes ☐ No

If yes, which one:

☐ Crop rotation practices

☐ Organic farming practices

☐ Reduction in pesticides & chemicals application

☐ Adopting water conserving practices

☐ Applying natural fertilizers & compost

☐ Biological control practices for insect/pest

☐ Other (please specify)

15. What factors do you consider when deciding which crop to grow? (Select all that apply)

☐ Market prices

☐ Input costs

☐ Crop rotation or intercropping benefits

☐ Soil health

☐ Weather/climate

☐ Access to resources (e.g., credit, seed, extension services)

☐ Labor availability

☐ Other (specify: ____)

16. Why did you decide to practice sustainable farming practices? (Select all that apply)

☐ Higher total yield

☐ Reduced pest and disease risk

☐ Improved soil health

☐ Higher-income potential

☐ Food security

☐ Availability of resources

☐ Environmental concerns

☐ Other (specify: ____)

17. What are the challenges you face with sustainable farming practices? (Select all that apply)

☐ Increased labor requirements

☐ Pest management difficulties

☐ Limited market access for sustainably produced rice

☐ Higher input costs

☐ Lack of knowledge/training on sustainable farming

☐ Weather/climate-related issues

☐ Other (specify: ____)

18. Have you received any support or training on sustainable farming practices?

☐ Yes ☐ No

If yes, who provided it? (Extension department, NGOs, research institute, private sector, etc.)

specify: ____

19. How do you consider adopting sustainable farming practices?

☐ Costly ☐ Cost effective

20. Do you have access to financial support (e.g., credit, subsidies) for adopting sustainable farming practices?

☐ Yes ☐ No

If yes, please describe the type of support you have received & financial institution (name of commercial bank, micro-finance bank, & NGO, etc):

____

21. How do you perceive the profitability of sustainable farming practices compared to conventional practices?

☐ More profitable; about _______ % higher

☐ Less profitable; about _______ % lower

☐ Neither profitable nor unprofitable

22. How do you perceive the risk of adopting sustainable farming practices?

☐ High ☐ Moderate ☐ Low ☐ None

23. What risks do you perceive associated to sustainable farming practices?

☐ Economical ☐ Socio-cultural ☐ Climatic & environmental ☐ Policy & institution

Section 3: Perceptions about Impact of SFP

24. Do you consider sustainable farming as environment friendly?

☐ Yes ☐ No

25. How important is it for you to protect the environment?

☐ Very important ☐ Somewhat important

☐ Not very important ☐ Not important at all

26. How concerned are you about the impacts of climate change on your farm?

☐ Very concerned ☐ Somewhat concerned

☐ Not very concerned ☐ Not concerned at all

27. How confident are you in your ability to adopt sustainable farming practices?

☐ Very confident ☐ Somewhat confident

☐ Not very confident ☐ Not confident at all

28. Would you recommend sustainable farming practices to other farmers?

☐ Yes ☐ No

29. Do you think the government should do more to promote sustainable farming practices?

☐ Yes ☐ No

If yes, what specific actions should the government take? (specify)

____

Section 4: Challenges for Adoption of SFP

30. Do you face barrier/s in adopting sustainable farming practices?

☐ Yes ☐ No

If yes specify please ____

31. Do you perceive a risk of adopting sustainable farming practices compared to conventional practices?

☐ Yes ☐ No

If yes specify please ____

32. Do you face challenges in accessing markets for sustainably produced rice?

☐ Yes ☐ No

If yes specify please ____

33. Does the lack of access to financial support (e.g., credit, subsidies) impact your ability to adopt sustainable farming practices?

☐ Yes ☐ No

If yes specify please ____

34. Do you face challenges in obtaining information/ training on sustainable farming practices?

☐ Yes ☐ No

If yes specify please ____

35. Does the lack of access to modern technologies impact your ability to adopt sustainable farming practices?

☐ Yes ☐ No

If yes specify please ____

36. Would incentives motivate you to adopt sustainable farming practices?

☐ Yes ☐ No

37. Do you think the government should do more to promote sustainable farming practices?

☐ Yes ☐ No

38. Do you believe the government should improve access to financial support (e.g., credit, subsidies) for adopting sustainable farming practices?

☐ Yes ☐ No

39. Do you think agricultural extension services can play a role in promoting sustainable farming practices?

☐ Yes ☐ No

40. Do you believe the government should improve access to markets for sustainably produced rice?

☐ Yes ☐ No

41. Do you need additional support or resources to adopt sustainable farming practices?

☐ Yes ☐ No

42. Do you think the government should improve access to training and education on sustainable farming practices?

☐ Yes ☐ No

43. Do you believe farmer cooperatives and associations can play a role in promoting sustainable farming practices?

☐ Yes ☐ No

44. Do you think the government should promote the adoption of sustainable farming practices through awareness campaigns?

☐ Yes ☐ No

45. Do you believe research institutions can play a role in developing and promoting sustainable farming practices?

☐ Yes ☐ No

Thank you for your participation!
